# Supplementary material for: Neutrophil Swarming in Damaged Tissue Is Orchestrated by Connexins and Cooperative Calcium Alarm Signals
Source: Curr Biol. 2020 Jul 20;30(14):2761–2776.e7. doi: 10.1016/j.cub.2020.05.030 (PMC7372224; doi:10.1016/j.cub.2020.05.030)
Supplement: Document S1. Figures S1–S7 [file mmc1.pdf]

Current Biology, Volume *30*

Supplemental Information

Neutrophil Swarming in Damaged Tissue Is Orchestrated by Connexins and Cooperative Calcium Alarm Signals

Hugo Poplimont, Antonios Georgantzoglou, Morgane Boulch, Hazel A. Walker, Caroline Coombs, Foteini Papaleonidopoulou, and Milka Sarris

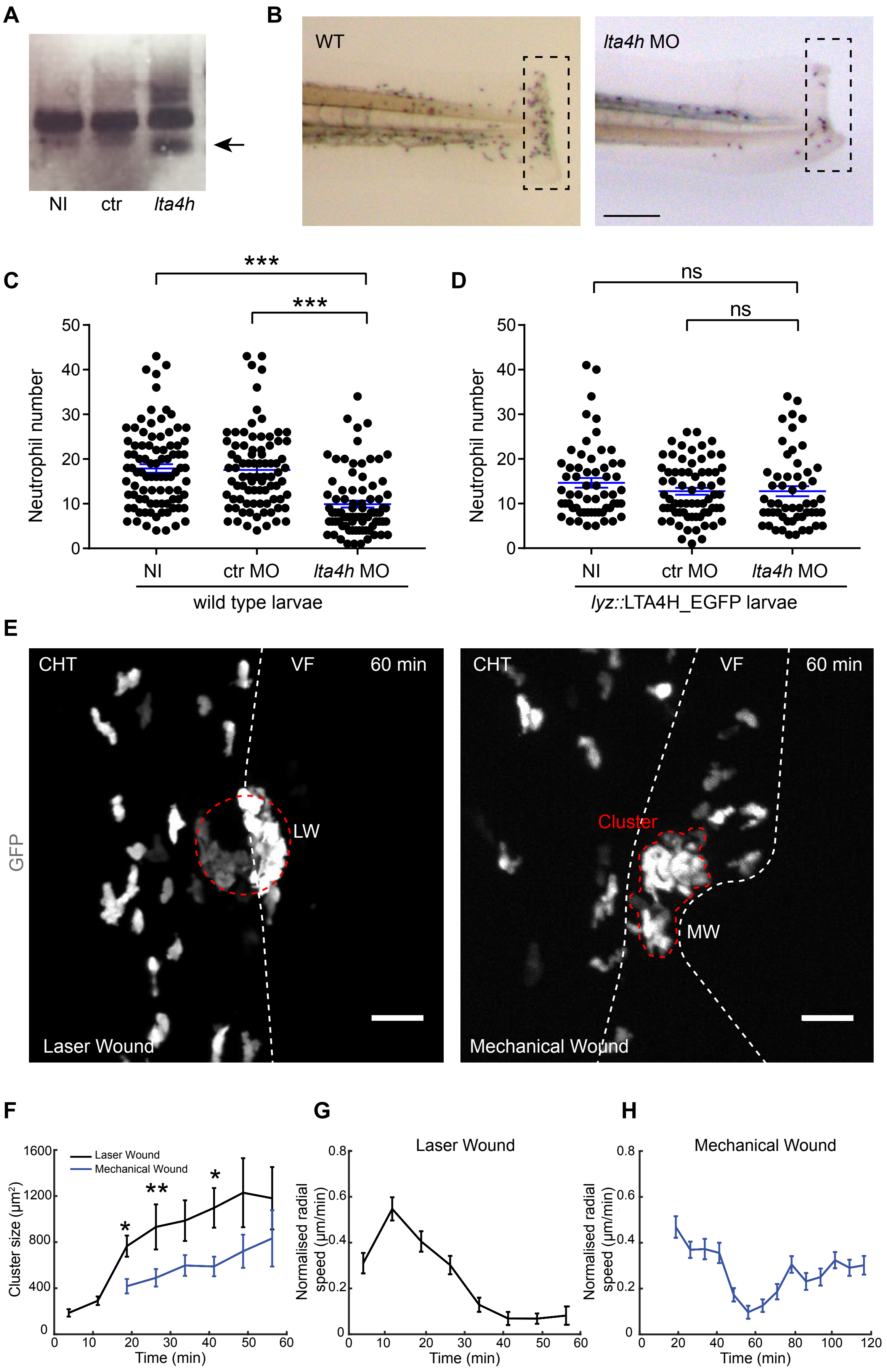

A
NI
ctr
lta4h
B
WT
lta4h MO
C
***
***
Neutrophil number
0
10
20
30
40
50
NI
ctr MO
lta4h MO
wild type larvae
D
ns
ns
Neutrophil number
0
10
20
30
40
50
NI
ctr MO
lta4h MO
lyz::LTA4H_EGFP larvae
E
GFP
CHT
VF
60 min
LW
Laser Wound
CHT
VF
60 min
Cluster
MW
Mechanical Wound
F
Laser Wound
Mechanical Wound
Cluster size (μm²)
0
400
800
1200
1600
*
**
*
Time (min)
0
10
20
30
40
50
60
G
Laser Wound
Normalised radial speed (μm/min)
0
0.2
0.4
0.6
0.8
Time (min)
0
10
20
30
40
50
60
H
Mechanical Wound
Normalised radial speed (μm/min)
0
0.2
0.4
0.6
0.8
Time (min)
0
20
40
60
80
100
120

Figure S1. Role of neutrophil biosynthesis of LTB4 and dynamics of neutrophil swarming in zebrafish wounds, related to Figure 1.

A RT-PCR of *lta4h* from mRNA extracted from 3 dpf wild type (AB) zebrafish. The arrow denotes the presence of an alternative transcript in larvae injected with a splice-blocking *lta4h* morpholino; NI = Non-injected, Ctr = Control morpholino, *lta4h* = injected with the splice-blocking *lta4h* morpholino.

B Sudan black staining of neutrophils in wild type (WT) and *lta4h* morpholino-injected 3dpf larvae amputated with a scalpel and fixed 3h post-wounding. The dotted lines represent the area in which neutrophils were counted. Scale bar = 100μm.

C, **D** Quantification of the total number of neutrophils recruited to wounds. NI = Non-injected, Ctr MO = Control morpholino, *lta4h* = injected with the splice-blocking *lta4h* morpholino. **C** 81-89 larvae per group pooled from 3 experiments. **D** 54-68 larvae per group pooled from 3 experiments. p value <0.0001. Kruskal-Wallis test with Dunn's post-test.

E Projections from two photon (left) and spinning disk confocal images (right) showing neutrophils from Tg(*mpx*:GFP)^{i114} larvae migrating from the caudal hematopoietic tissue (CHT) towards a laser wound (LW; dotted line) at the ventral fin-CHT boundary (VF/CHT) or mechanical ventral fin wound (MW) respectively. The red dotted line indicates area occupied by the neutrophil cluster. Scale bar = 25μm.

F Evolution of cluster size over time post wounding for neutrophils in Tg(*mpx*:GFP)^{i114} larvae. n=4 larvae with laser wound, n=14 larvae with mechanical fin wound from 2 and 7 experiments respectively. Mann Whitney test.

G Evolution of neutrophil radial speed over time post laser wounding (LW) for neutrophils in Tg(*mpx*:GFP)^{i114} or Tg(*lyz*:GCamp6F) larvae. Instantaneous speed values for individual neutrophils were divided by the mean instantaneous speed value of the corresponding embryo to normalise differences in speed across embryos (see also methods). Error bars represent

standard error of the mean from data binned every 7.5 min. n=1201-1719 cell-steps per bin from n=10 larvae.

H Evolution of neutrophil radial speed over time post mechanical fin wounding for neutrophils in Tg(*mpx*:GFP)^{i114} or Tg(*lyz*:GCamp6F) larvae. Error bars represent standard error of the mean from data binned every 7.5 min from n=1248-1800 cell-steps per bin from 14 larvae.

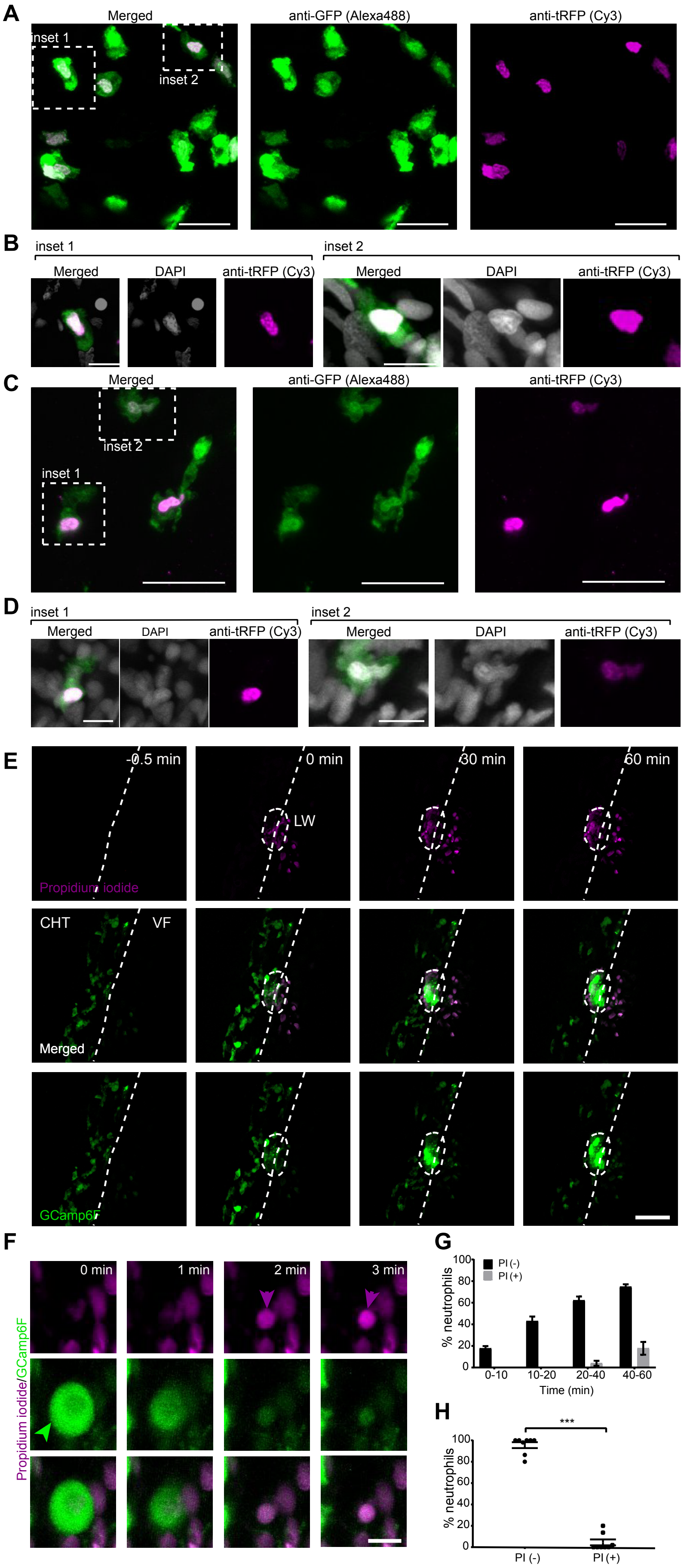

A
Merged
anti-GFP (Alexa488)
anti-tRFP (Cy3)
inset 1
inset 2
B
inset 1
inset 2
Merged
DAPI
anti-tRFP (Cy3)
C
Merged
anti-GFP (Alexa488)
anti-tRFP (Cy3)
inset 2
inset 1
D
inset 1
inset 2
Merged
DAPI
anti-tRFP (Cy3)
E
-0.5 min
0 min
30 min
60 min
LW
Propidium iodide
CHT
VF
Merged
GCamp6F
F
0 min
1 min
2 min
3 min
Propidium iodide/GCamp6F
G
PI (-)
PI (+)
% neutrophils
0-10
10-20
20-40
40-60
Time (min)
H
***
% neutrophils
PI (-)
PI (+)

Figure S2. Nuclear localisation of 5-LO in zebrafish neutrophils and kinetics of neutrophil death in the first hour post-wounding, related to Figures 2 and 3

A-D Laser scanning confocal projections of Tg(*mpx*:GFP)^{i114}xTg(*lyz*:tRFP-5LO) larvae stained with antibodies against GFP (green) and tRFP (magenta) and co-labelled with nuclear DAPI staining (white) A and C represent examples from two different larvae (Scale bar=25µm). B and D represent zoomed-in images corresponding to insets in A and C respectively (Scale bar=10µm).

E Time-lapse series of two-photon confocal projections images of neutrophils Tg(*lyz*:GCamp6F) larvae (green) incubated in PI (magenta). Time post-wounding is indicated in minutes. Dotted line indicates laser wound (LW). CHT: caudal hematopoietic tissue. VF: ventral fin. Scale bar=50µm.

F Zoomed in example of an apoptotic neutrophil. The green arrow indicates a neutrophil with an apoptotic shape in the GCamp6F channel. The magenta arrows indicate the nucleus of this neutrophil subsequently up taking PI. Scale bar = 10µm.

G,**H** Percentage of dead or alive neutrophils over time (**G**) or averaged through the first hour post-wounding (**H**). Data are means of 8 larvae in 7 independent experiments. Mann-Whitney test.

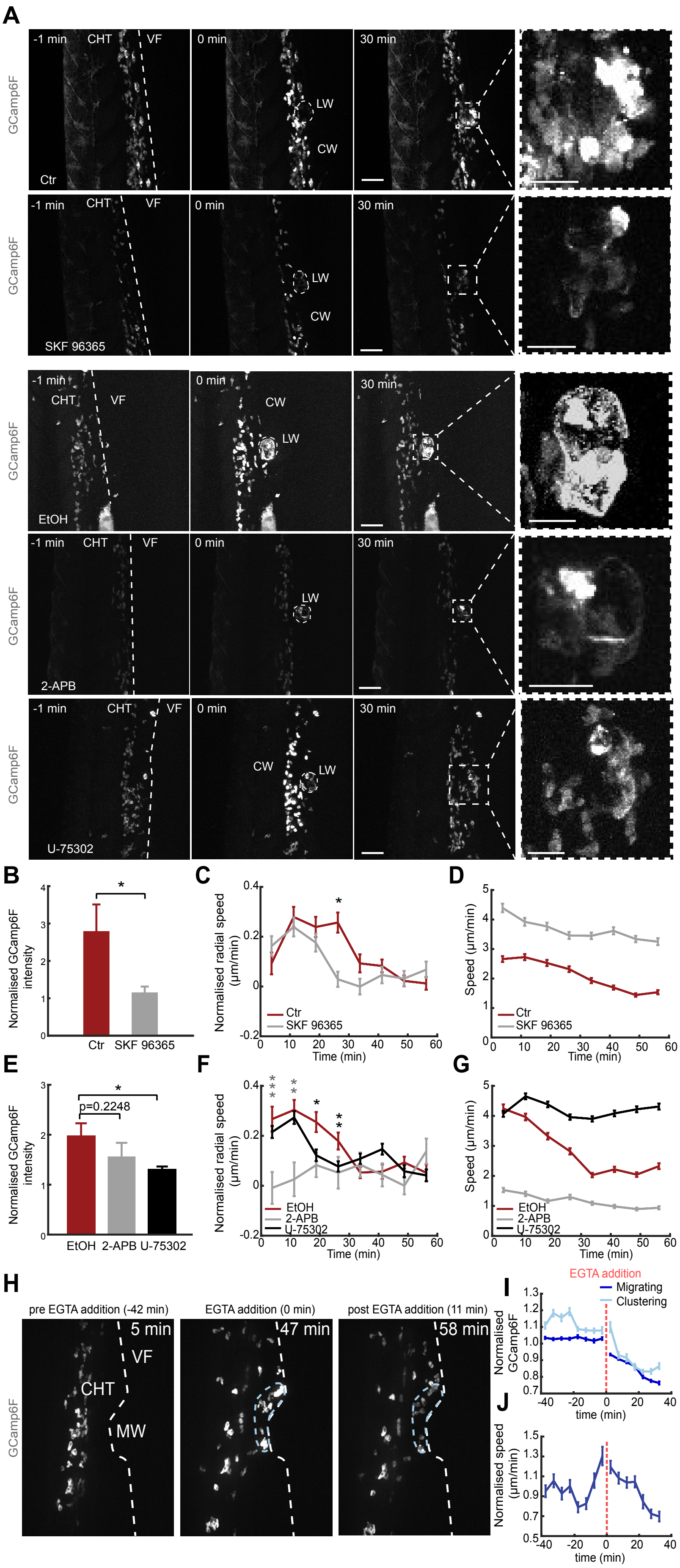

A
-1 min
CHT
VF
0 min
LW
CW
30 min
GCamp6F
Ctr
SKF 96365
EtOH
2-APB
U-75302
B
C
D
Normalised GCamp6F intensity
Ctr
SKF 96365
Normalised radial speed (µm/min)
Time (min)
Speed (µm/min)
E
F
G
p=0.2248
EtOH
2-APB
U-75302
H
pre EGTA addition (-42 min)
EGTA addition (0 min)
post EGTA addition (11 min)
5 min
47 min
58 min
MW
I
EGTA addition
Migrating
Clustering
Normalised GCamp6F
time (min)
J
Normalised speed (µm/min)

Figure S3. Effect of SKF96365, 2-APB, U-75302 and EGTA on neutrophil dynamics, related to Figure 4

A Time-lapse sequence of two-photon confocal image projections showing neutrophils in Tg(*lyz*:GCamp6F)xTg(*lyz*:tRFP-5LO) larvae, without treatment (ctr) or in the presence of 20 μM SKF96365, 25 μM 2-APB, 3 μM U-75302 (and control dilutions of Ethanol to be compared with the latter two). Scale bars = 50μm and 20μm, for zoomed-out and zoomed-in images respectively. CW: calcium wave. Time after laser wounding (LW) is shown in minutes. CHT: caudal hematopoietic tissue. VF: ventral fin.

B Normalised GCamp6F levels in control or SKF96365-treated larvae. n=5 control, n=4 SKF96365-treated larvae from 3 experiments. GCamp6F intensity was normalised as in Figure 1D. Mann-Whitney test.

C-D Neutrophil radial speed (C) or speed (D) over time post-laser wounding in control or SKF96365-treated larvae. n=1226-1527 cell-steps per bin from 5 control larvae and n=968-1219 cell-steps per bin from 4 SKF96365-treated larvae from 3 experiments. Mann-Whitney test.

E Normalised GCamp6F levels in Ethanol control, 2-APB and U-75302-treated larvae. n=5 control, n=4 2-APB-treated larvae and n=8 U-75302-treated larvae from 2, 2 and 3 independent experiments respectively. One-way ANOVA with Dunnet's multiple comparison post-test.

F-G Neutrophil radial speed (F) or speed (G) over time post-laser wounding in Ethanol control, 2-APB and U-75302-treated larvae. n=1464-1743 cell-steps per bin from 5 control larvae, n=679-771 cell-steps per bin from 4 2-APB-treated larvae and n=2712-3135 cell-steps per bin from 8 U-75302-treated larvae from 2, 2 and 3 independent experiments respectively. Kruskal-Wallis with Dunn's multiple comparison test (black stars; ctr vs U-75302, grey stars; ctr vs 2-APB).

H Spinning-disk confocal projection images from neutrophils in Tg(*lyz*:GCamp6F)xTg(*lyz*:tRFP-5LO) post-mechanical fin wound (MW) and EGTA treatment. Time after wounding is indicated within images. Time after EGTA addition is indicated above. The light blue dotted line indicates area occupied by the neutrophil cluster. Scale bar = 50µm.

I Normalised GCamp6F levels in clustering cells (light blue line; corresponding to cells within the wound area occupied by clustering neutrophils shown in J) or migrating cells (dark blue line; corresponding to cells outside the wound area) over time. Red dotted line indicates time of EGTA addition. n=288-810 cell-steps per bin for migrating cells, n=40-144 cell-steps per bin for clustering cells from 5 larvae in 2 independent experiments.

J Normalised neutrophil speed in migrating cells over time. Red dotted line indicates time of EGTA addition. n=170-677 cell-steps per bin from 5 larvae in 2 independent experiments.

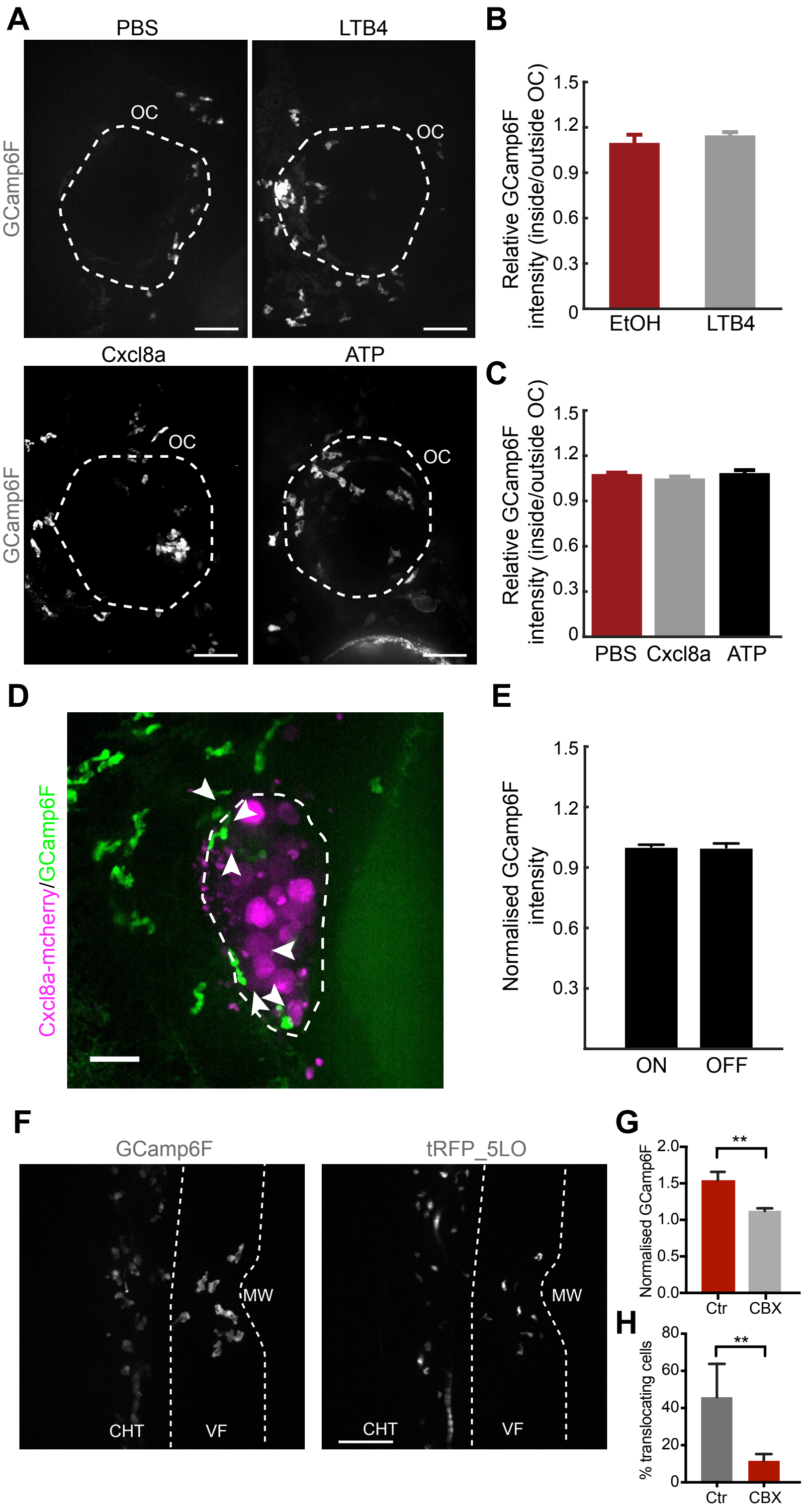
A
PBS
LTB4
OC
GCamp6F
Cxcl8a
ATP
B
Relative GCamp6F intensity (inside/outside OC)
EtOH
LTB4
C
PBS
Cxcl8a
ATP
D
Cxcl8a-mchery/GCamp6F
E
Normalised GCamp6F intensity
ON
OFF
F
tRFP_5LO
CHT
VF
MW
G
Normalised GCamp6F
Ctr
CBX
**
H
% translocating cells

Figure S4. Effect of chemoattractant injection or transplantation of chemokine-secreting cells on neutrophil calcium dynamics, related to Figures 4 and 5

A Confocal spinning disk projection images of neutrophils in Tg(*lyz*:Gcamp6F) around the otic cavity (OC) of 3 dpf larvae 50 min after local injection of 30 nM LTB4, 30 nM Cxcl8a, 200 µM non-hydrolysable ATP-γS and control and PBS control. The otic cavity is delimited by dotted lines. Scale bar = 50µm.

B and **C** Relative GCamp6F levels in Ethanol (EtOH) control, PBS control, LTB4-injected Cxcl8a-injected and ATP-injected Tg(*lyz*:GCamp6F) larvae. Levels of GCamp6F in neutrophils within the otic cavity (OC) are expressed relative to the levels of cells outside the wound cavity in the first image of the movie. n=5 PBS, n=5 EtOH, n=4 LTB4, n=5 ATP and n=5 Cxcl8a larvae from 2, 3, 2, 2 and 2 independent experiments respectively.

D Confocal spinning disk projection images of neutrophils in Tg(*lyz*:Gcamp6F) 3 dpf larvae (in green) moving around a transplant of HEK293T cells expressing Cxcl8a-mCherry (magenta). The transplantation zone is delimited by dotted lines and neutrophils within this area are indicated with white arrows. Scale bar = 25µm.

E Quantification of GCamp6F intensity in neutrophils on the Cxcl8a transplant (ON) and beyond (OFF). Data are means of n=4 independent embryos.

F Spinning-disk confocal projection images of neutrophils in Tg(*lyz*:GCamp6F)xTg(*lyz*:tRFP-5LO) larvae 90 minutes after mechanical ventral fin wound (MW) in the presence of 50 µM CBX. Representative control shown in Figure 4F, for comparison. MW: mechanical wound. CHT: caudal hematopoietic tissue. VF: ventral fin. Scale bar = 50µm.

G Mean normalised GCamp6F intensity in Tg(*lyz*:GCamp6F)xTg(*lyz*:tRFP-5LO) larvae treated or not with CBX. GCamp6F intensity was normalised as in Figure 1D and E. Data are

means from n=9 control larvae and n= 5 CBX-treated larvae from 3 and 2 imaging sessions respectively. Mann-Whitney test.

H Percent of translocating neutrophils out of all neutrophils recruited into the ventral fin over 90 min of imaging starting 10 min post-wounding. Data are means of n=7 control larvae and n=5 CBX-treated larvae from 3 and 2 imaging sessions respectively. Mann-Whitney test.

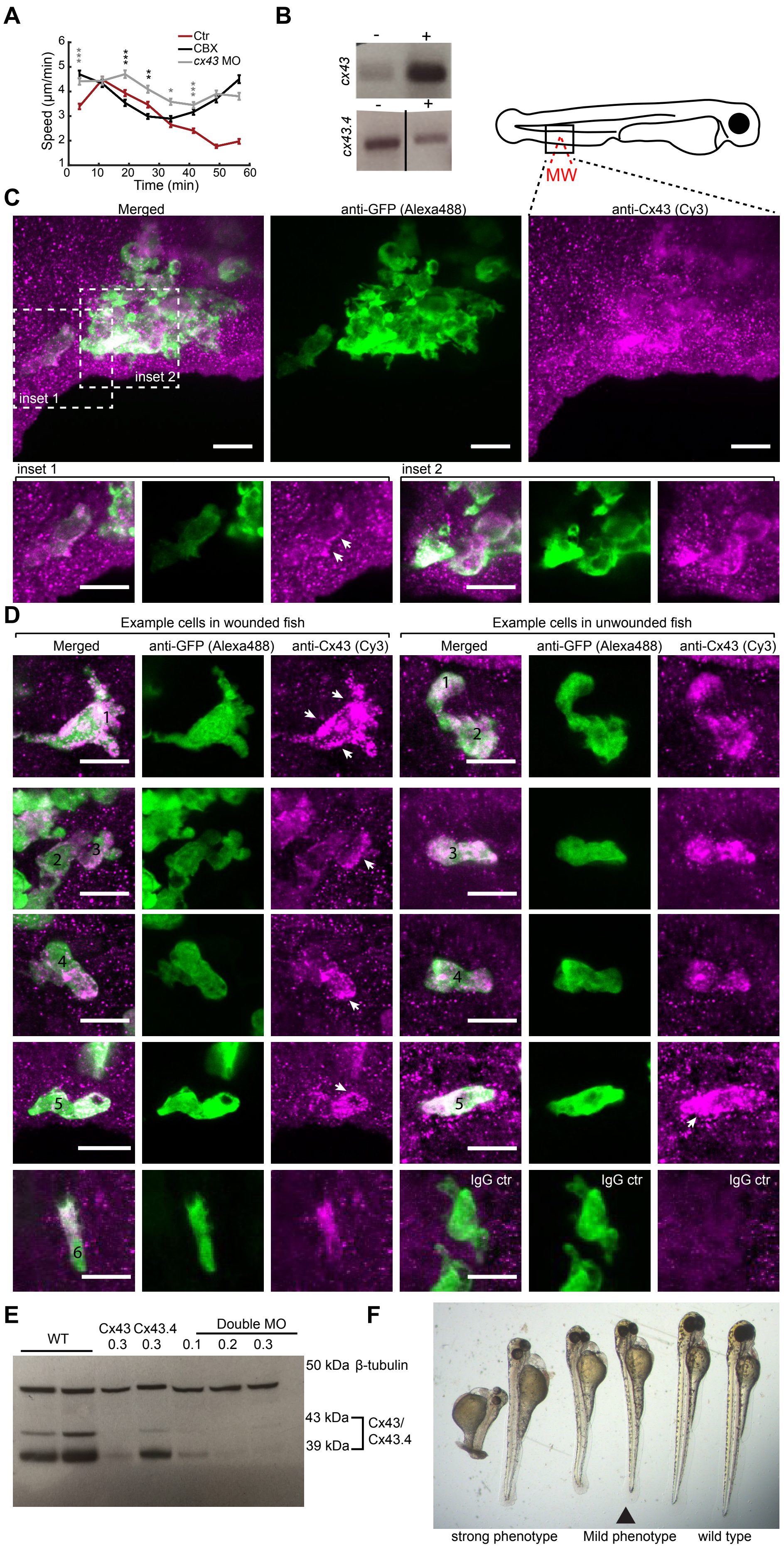
A
B
C
D
E
F
Ctr
CBX
cx43 MO
Speed (µm/min)
Time (min)
cx43
cx43.4
MW
Merged
anti-GFP (Alexa488)
anti-Cx43 (Cy3)
inset 1
inset 2
Example cells in wounded fish
Example cells in unwounded fish
IgG ctr
WT
Cx43 Cx43.4
Double MO
50 kDa β-tubulin
43 kDa
39 kDa
Cx43/
Cx43.4
strong phenotype
Mild phenotype
wild type

Figure S5. Neutrophil Cx43 expression and knockdown in zebrafish larvae and effects on neutrophil dynamics, related to Figure 5

A Neutrophil speed over time post-laser wounding in control, CBX-treated and *cx43* MO-treated larvae from Tg(*mpx*:GFP)^{i114} and Tg(*lyz*:GCamp6F) larvae. n=1201-1719 cell-steps per bin from 12 control, n=1268-1535 cell-steps per bin from 8 CBX-treated and n=1308-1554 cell-steps per bin from 11 *cx43* MO-injected larvae from 8, 3 and 4 experiments respectively. Mann-Whitney test (grey stars indicate significance for both Ctr vs CBX and Ctr vs *cx43* MO; black stars indicate significance for just Ctr vs CBX).

B RT-PCR for *cx43* and *cx43.4* expression from cDNA samples of FACS-sorted GFP^{+} (+) or GFP^{-} cells (-) from Tg(*mpx*:GFP)^{i114} 4.5 dpf larvae.

C-D Laser scanning confocal projections of Tg(*mpx*:GFP)^{i114} larvae stained with antibodies against GFP (green) and Cx43/Cx43.4 (magenta). **C** represents example of a cluster overview from one larva (Scale bar = 25µm) with insets showing a closer view of a subset of clustering cells (Scale bar = 10µm). **D** shows additional examples of neutrophils from 5 wounded and 4 unwounded larvae stained with anti-Cx43 and from 1 wounded larva stained with IgG ctr. Scale bar=10µm. Neutrophils are marked with a number. In the examples from wounded larvae, cells 1-5 are at the wound margin and cell 6 is in the CHT. In the examples of unwounded larvae, cells 1-5 are in the CHT. The IgG control example is at the wound margin of a wounded larva. Cartoon shows area of mechanical wounding (MW) and imaging. Arrows indicate example regions with Cx43 stain on the periphery of the neutrophil.

E Western Blot showing the expression of Cx43 and Cx43.4 in 3dpf larvae of wild type AB (WT) and morphant zebrafish larvae. Amount injected for each morpholino in pmol is shown.

F Image of different phenotypes of 3dpf zebrafish larvae injected with *cx43*/*cx43.4* combination MOs. The black arrow indicates the mildest phenotype with detectable difference in eye size that was selected for subsequent experiments.

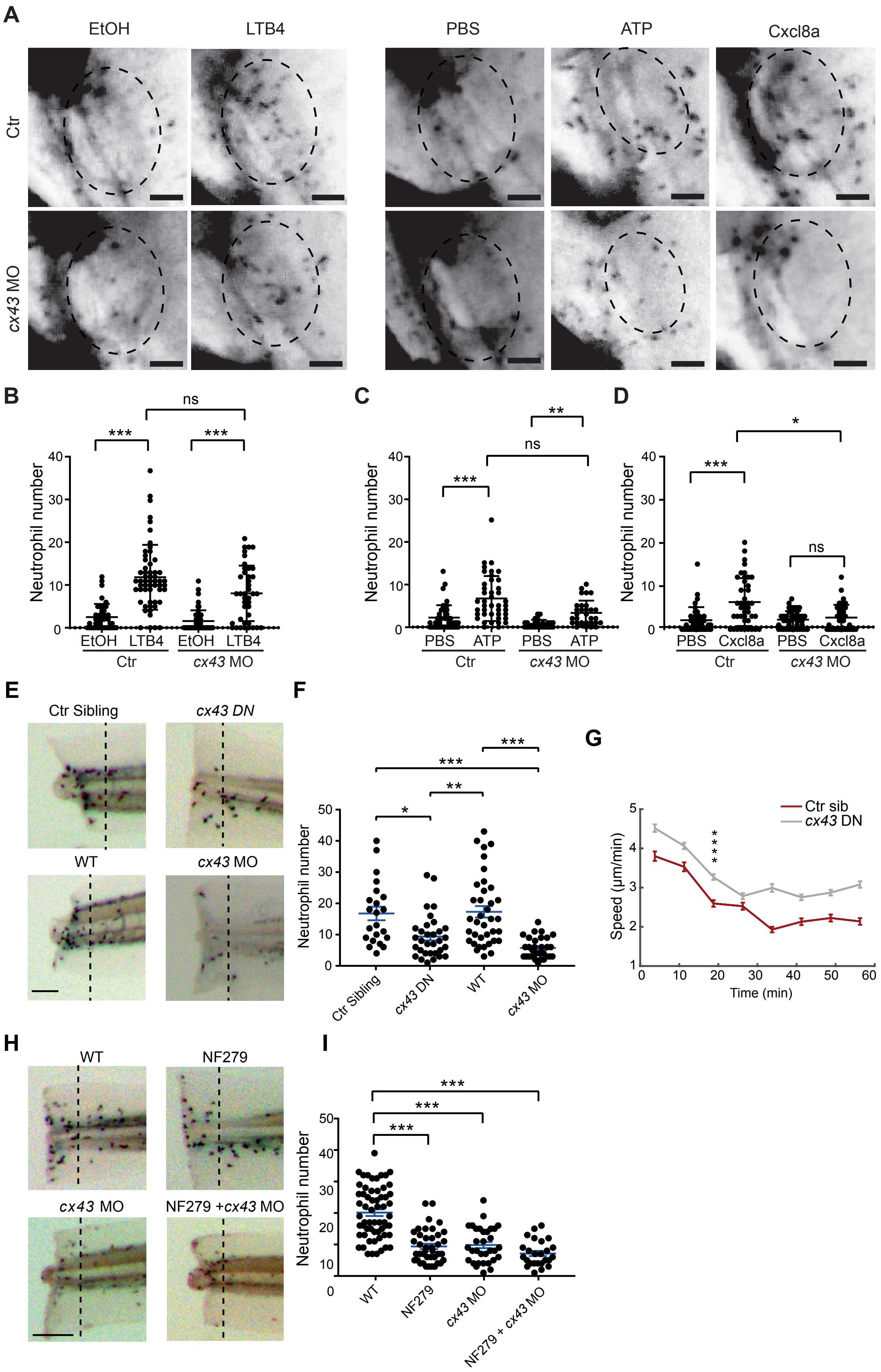
A
EtOH
LTB4
PBS
ATP
Cxcl8a
Ctr
cx43 MO
B
C
D
Neutrophil number
ns
***
**
*
EtOH LTB4 EtOH LTB4
PBS ATP PBS ATP
PBS Cxcl8a PBS Cxcl8a
Ctr
cx43 MO
E
Ctr Sibling
cx43 DN
WT
cx43 MO
F
Ctr Sibling
cx43 DN
WT
cx43 MO
G
Ctr sib
cx43 DN
Speed (µm/min)
Time (min)
H
WT
NF279
cx43 MO
NF279 +cx43 MO
I
WT
NF279
cx43 MO
NF279 + cx43 MO

Figure S6. Recruitment of neutrophils in response to chemoattractant injection and effect of Cx43 inhibition on wound recruitment with or without co-inhibition of ATP signalling, related to Figure 5

A Sudan black staining of neutrophils in transgenic wild type AB larvae 45 min following injection of Ethanol control, PBS control, 30 nM LTB4, 30 nM Cxcl8a and 200 µM non-hydrolysable ATP-γS in the otic vesicle, with or without *cx43* MO injection. The dotted lines represent the periphery of the cavity where neutrophils were counted. Scale bar = 50µm.

B-D Number of neutrophils recruited to the otic vesicle at 45 min. n=30-56 larvae per condition, across 2 independent experiments. Kruskal-Wallis test with Dunn's post-test.

E Sudan black staining of neutrophils in transgenic Tg(*lyz:cx43DN*-T2A-mCherry) larvae (*cx43DN*) and their negatively screened siblings (Sibling) or larvae injected with *cx43/cx43.4* combination of morpholinos (*cx43* MO), fixed at 3h after wounding. The dotted lines represent the area in which neutrophils were counted. Scale bar = 50µm.

F Number of neutrophils recruited to wounds in the different conditions pooled from three independent experiments. *cx43 DN,* n=32 larvae; negative siblings (Ctr sibling), n=22 larvae; WT, n=37 larvae; *cx43* MO, n=30 larvae. Kruskal-Wallis test with Dunn's post-test.

G Neutrophil speed over time post-laser wounding in Tg(*lyz*:GCamp6F)xTg(*lyz*:*cx43DN*-T2A-mCherry) zebrafish larvae, positive (*cx43* DN) or negative for the Cx43 DN-T2A-mCherry transgene (control siblings: Ctr sib). n=1330-1602 cell-steps per bin from 5 control siblings, n=2573-3050 cell-steps per bin from 7 *Cx43* DN larvae from 3 experiments. Mann-Whitney test.

H Sudan black staining of neutrophils in wild type larvae injected or not with *cx43/cx43.4* combination of morpholinos (*cx43* MO) and treated or not with 10µM NF279 and fixed 3h after wounding. Scale bar =100µm.

I Quantification of neutrophil number at wounds in the different conditions. Data pooled from 2 independent experiments. WT (non-injected and not treated with drug), n=60 larvae; NF279, n=40; cx43 MO, n=32; cx43 MO with NF279, n=25. One-way ANOVA with Tukey's multiple comparison test.

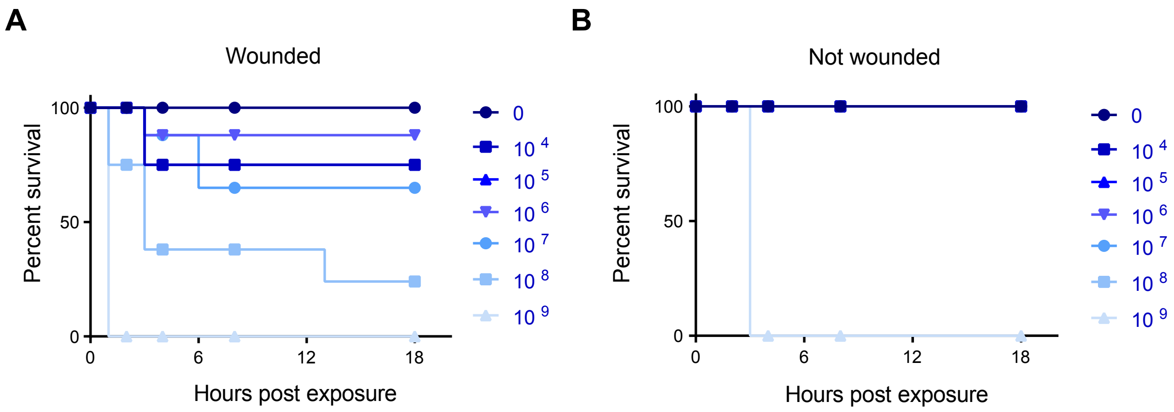

Figure S7. Zebrafish tail fin wound colonisation assay with *P. aeruginosa*, related to Figure 7

A 3 dpf larvae were injured at the tail fin with a scalpel in the presence of titrating doses of *P. aeruginosa* in the bath. Concentration-dependent effects on survival in wounded larvae are shown.

B Same titration in non-wounded larvae. Injury-independent death is observed only at doses below 10^9 CFU/ml. Data represent one experiment with 20 larvae per condition.
