# Supplementary material for: Neutrophil Swarming in Damaged Tissue Is Orchestrated by Connexins and Cooperative Calcium Alarm Signals
Source: Curr Biol. 2020 Jul 20;30(14):2761–2776.e7. doi: 10.1016/j.cub.2020.05.030 (PMC7372224; doi:10.1016/j.cub.2020.05.030)
Supplement: Document S2. Article plus Supplemental Information [file mmc9.pdf]

Current Biology

Article

Neutrophil Swarming in Damaged Tissue Is Orchestrated by Connexins and Cooperative Calcium Alarm Signals

Graphical Abstract

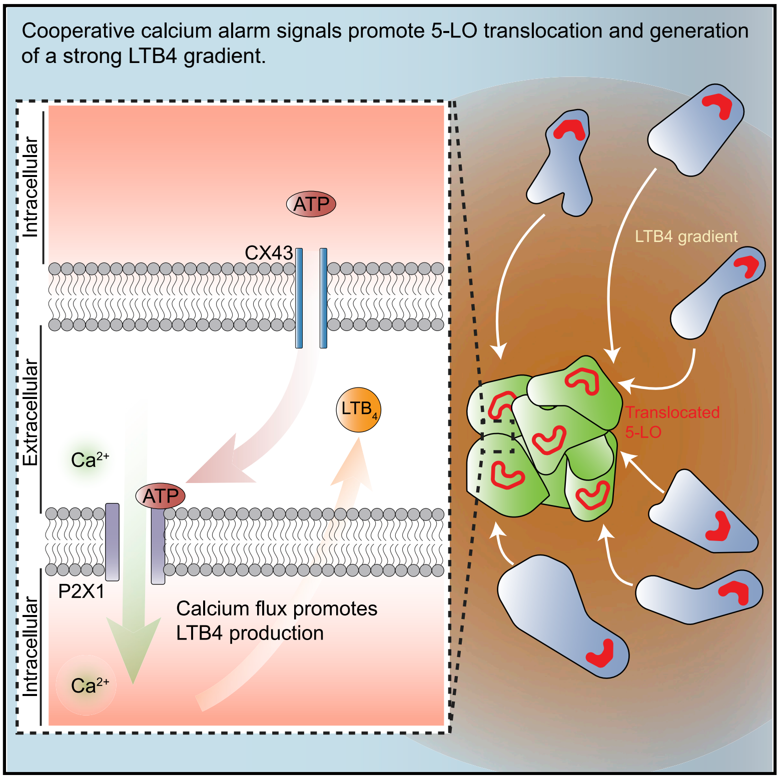

Highlights

- Calcium alarm signals in neutrophil clusters locally promote attractant synthesis
- Calcium alarm signals are dependent on ATP sensing and contact with necrotic tissue
- Clustering neutrophils initiate and propagate calcium alarm signals via Cx43 channels
- Neutrophil swarms and Cx43 restrict wound colonization by opportunistic bacteria

Authors

Hugo Poplimont, Antonios Georgantzoglou, Morgane Boulch, Hazel A. Walker, Caroline Coombs, Foteini Papaleonidopoulou, Milka Sarris

Correspondence

ms543@cam.ac.uk

In Brief

Poplimont et al. show that frontline neutrophils clustering at wound sites centrally coordinate damage sensing, calcium alarm signals, and the escalation of the inflammatory response. Intracluster coordination of signaling requires connexins and leads to dense swarms, which protect the breached tissue from opportunistic pathogens.

Poplimont et al., 2020, Current Biology *30*, 2761–2776
July 20, 2020 © 2020 The Authors. Published by Elsevier Inc.
https://doi.org/10.1016/j.cub.2020.05.030

CellPress

Current Biology

CellPress
OPEN ACCESS

Article

Neutrophil Swarming in Damaged Tissue Is Orchestrated by Connexins and Cooperative Calcium Alarm Signals

Hugo Poplimont,[1] Antonios Georgantzoglou,[1] Morgane Boulch,[1] Hazel A. Walker,[1] Caroline Coombs,[1] Foteini Papaleonidopoulou,[1] and Milka Sarris[1,2,*]

[1]Department of Physiology, Development and Neuroscience, University of Cambridge, Downing Site, Cambridge CB2 3DY, UK

[2]Lead Contact

*Correspondence: ms543@cam.ac.uk

https://doi.org/10.1016/j.cub.2020.05.030

SUMMARY

Neutrophils are major inflammatory cells that rapidly infiltrate wounds to provide antimicrobial functions. Within the damaged tissue, neutrophil migration behavior often switches from exploratory patrolling to coordinated swarming, giving rise to dense clusters that further disrupt tissue architecture. This aggregation response is self-organized by neutrophil paracrine chemoattractant signaling (most notably of the inflammatory mediator leukotriene B4 [LTB4]). The coordination mechanism and possible evolutionary benefits of neutrophil swarms are elusive. Here, we show that neutrophil swarms require mutual reinforcement of damage signaling at the wound core. New biosensors and live imaging in zebrafish revealed that neutrophil chemoattractant synthesis is triggered by a sustained calcium flux upon contact with necrotic tissue that requires sensing of the damage signal ATP. This "calcium alarm" signal rapidly propagates in the nascent neutrophil cluster in a contact-dependent manner via connexin-43 (Cx43) hemichannels, which are mediators of active ATP release. This enhances chemoattractant biosynthesis in the growing cluster, which is instrumental for coordinated motion and swarming. Inhibition of neutrophil Cx43 compromises clearance of wound-colonizing *P. aeruginosa* bacteria and exacerbates infection-induced morbidity. Thus, cooperative production of alarm signals among pioneer clustering neutrophils fuels the growth of dense antimicrobial cell masses that effectively seal off breached tissue barriers from opportunistic pathogens.

INTRODUCTION

Tissue damage triggers rapid recruitment of immune cells, with neutrophils as prime infiltrators [1, 2]. This migratory response marks the onset of inflammation, which is essential for protecting the breached tissue from infection while the slow process of tissue repair unfolds. Neutrophils are instrumental for killing bacterial pathogens through phagocytosis, release of proteolytic enzymes, and reactive radicals [1]. However, prolonged neutrophil residence can cause collateral tissue damage, perpetuate inflammation, and delay tissue repair and restoration of homeostasis [1]. Chronic inflammation forms the basis of numerous diseases and can also be co-opted by cancer cells to favor tumor growth and metastasis [3, 4]. Tuning neutrophil accumulation to desirable levels is thus an important biomedical target, yet our basic understanding of how this response naturally escalates under physiological conditions remains limited.

Interestingly, although the initial steps in neutrophil recruitment are driven by extrinsic cues, the escalation phase of the response is largely self-organized. Tissue injury results in local release of primary damage cues (damage-associated molecular patterns or DAMPs) from necrotic cells, including ATP or formyl peptides, which are normally not present in the extracellular environment [1, 2, 5]. To a certain extent, these primary signals may act directly as chemoattractants by signaling through corresponding G-protein-coupled receptors (GPCRs) [5]. Beyond this, DAMPs and other physiological stresses cause secondary production of chemoattractants by local tissue cells, including chemokines or arachidonic acid metabolites [1, 6, 7]. Altogether, this cocktail of attractants promotes exit of neutrophils from the blood (extravasation) and biased directional motion (chemotaxis) toward the site of injury within minutes. Thereafter, neutrophil behavior can switch from mere chemotaxis to highly coordinated and unidirectional motion that culminates in dense clusters at the wound core [8–10]. This so-called "swarming" or "aggregation" behavior is self-organized, as it relies on paracrine release of the lipid attractant leukotriene B4 (LTB4) by neutrophils [6, 8]. The decision to release chemoattractant is thus critical for the ultimate scale of the response. However, it remains unclear how neutrophil activation and chemoattractant synthesis might be coordinated in individual neutrophils.

Coordination of signaling has been found to play a role in reminiscent phenomena in unicellular organisms. Upon starvation, unicellular slime mold amoebae aggregate into a multicellular

This is an open access article under the CC BY license (http://creativecommons.org/licenses/by/4.0/).

migratory slug capable of seeking nutrients [11]. This is driven by initial production of the chemoattractant cyclic AMP (cAMP) in a single amoeba, which triggers further cAMP release in nearby cells, resulting in traveling waves of attractant. Coordination of this response toward a single center point requires periodic and polarized emission of signal [11, 12]. By analogy, neutrophils may require specific signaling dynamics to trigger swarming [6, 9, 13]. However, how the attractant production might be triggered and coordinated across single neutrophils *in situ* is unknown. Recent evidence shows that macrophages can prevent swarming by cloaking the wound area, suggesting neutrophil access to the necrotic site is important [14]. Another interesting clue is that a critical threshold of initial clustering at the site of damage correlates with subsequent swarming [15]. However, directly relating these observations to neutrophil activation and chemoattractant synthesis has been hampered by the lack of tools to monitor the relevant signals *in vivo*.

Here, we take advantage of the genetic and imaging amenability of zebrafish, in which neutrophil swarming is conserved. We visualized the intracellular events leading to LTB4 synthesis in individual neutrophils. This revealed that activation of LTB4 biosynthesis preferentially occurs in clustering neutrophils at the wound core rather than individual migrating cells. This activation is associated with distinct calcium alarm signals that are triggered by contact with necrotic tissue and propagated among clustering neutrophils. Formation and intracluster propagation of these calcium alarm signals are dependent on connexin-43 (Cx43) hemichannels, which allow ATP release from live neutrophils, leading to autocrine and juxtacrine amplification of damage signaling. This communication coordinates and amplifies calcium fluxes in the cluster, locally promoting attractant production. Inhibition of connexin-mediated communication suppresses swarming and increases wound susceptibility to infection, suggesting that neutrophil swarming may have evolved as a beneficial host defense mechanism.

RESULTS

Distinct Calcium Signals in Clustering Neutrophils

Neutrophil swarming is conserved in zebrafish, a model ideally suited for imaging and genetic manipulation [9, 16]. To establish the role of neutrophil LTB4 production in this model, we generated a transgenic zebrafish line, Tg(*lyz:lta4h-E*GFP), expressing leukotriene A4 hydrolase (LTA4H), an enzyme that catalyzes the conversion of LTA4 into LTB4 [17], the final step in LTB4 biosynthesis. We used a previously validated translation-blocking morpholino [18] to suppress *lta4h* expression, and this led to reduced neutrophil accumulation in wounds (Figures S1A–S1C). In contrast, *lta4h* knockdown did not affect neutrophil accumulation in wounds of Tg(*lyz:lta4h*-EGFP) larvae (Figure S1D). This confirmed that neutrophil-derived LTB4 drives neutrophil accumulation at wounds, as observed in mammalian systems.

We next characterized the dynamics of neutrophil swarming in two types of wound models. Because swarms are more likely to occur under high neutrophil density [8, 15, 19], we visualized the behavior of neutrophils after acute laser wound injury at a site rich in neutrophils, the caudal hematopoietic tissue (CHT), using two-photon ablation (Figures 1A and S1E; Video S1). Within 5 min, neutrophils began migrating to the wound in a highly directional and coordinated manner, forming clusters at the wound core by 20 min (Figure S1F; Video S1). To quantify this, we measured the radial speed of neutrophils over time, which reflects the level of coordination of migration [8]. When cells move in synchrony in the same direction, the amplitude of radial speed of the population is high. Accordingly, we detected a marked wave of synchronous directional motion, peaking at 15 min post-wounding in laser wounds (Figure S1G; Video S1). This time course is comparable with the evolution of neutrophil radial speed in mouse laser wounds [8]. We compared this to neutrophil responses in mechanical wounds, executed at the ventral fin nearby the CHT. In this model, neutrophil swarms could be imaged no earlier than 15 min post-wounding and showed lower magnitude of clustering and more variable peaks of radial speed (Figures S1E–S1H; Video S1). We prioritized the laser wound assay to capture the swarm initiation and exploit the faster and less variable cell dynamics.

LTB4 production requires calcium-dependent translocation of biosynthetic enzymes to membrane compartments where lipid metabolism takes place [20]. Intracellular calcium dynamics have been observed in zebrafish epithelial cells [7] and neutrophils migrating in a solitary manner [21], but not in swarming neutrophils. To characterize this, we generated a transgenic line expressing a sensitive calcium indicator, GCamp6F [22], in neutrophils, hereafter referred to as Tg(*lyz:*GCamp6F) (Figure 1B). We visualized the behavior of Tg(*lyz:*GCamp6F) neutrophils after acute laser wound injury and observed three distinct signals on the basis of GCamp6F fluorescence intensity (Figure 1C; Video S2): first, a brief, tissue-wide calcium wave immediately after wounding that dissipated within 30 s (Figures 1C and 1D), an anticipated transient response of tissue to injury [7, 23]. Subsequently, neutrophils in the CHT began migrating toward the wound and showed dynamic local fluctuations in calcium intensity (Video S2). In contrast, upon arrival at the wound core, neutrophils underwent a whole-cell, sustained calcium flux concomitant with clustering, which was of markedly higher amplitude than the low-level fluctuations in migrating neutrophils (Figure 1E; Video S2). A similar pattern of calcium signals was observed in smaller scale wounds (Video S2). Strikingly, the calcium fluxes rapidly propagated across clustering neutrophils, giving rise to a cellular mass with relatively sustained calcium signaling (Video S2). Quantification showed that the mean calcium intensity in clustering cells was sustained at high levels throughout the first hour post-wounding (Figure 1E). Moreover, calcium intensity showed a positive association with cluster size, as solitary cells had lower calcium levels than clustering cells (Figures 1F and 1G). Altogether, this evidence revealed distinct types of calcium signals in migrating cells versus clustering neutrophils.

Activation of LTB4 Biosynthesis Occurs Preferably in Calcium-Fluxing Neutrophils within Clusters

The discovery of distinct types of calcium signals in swarming neutrophils prompted us to investigate which of these are consequential on LTB4 biosynthesis. The rate-limiting step in LTB4 biosynthesis is the translocation of 5-lipoxygenase (5-LO or ALOX5) to the nuclear envelope membrane, where it converts

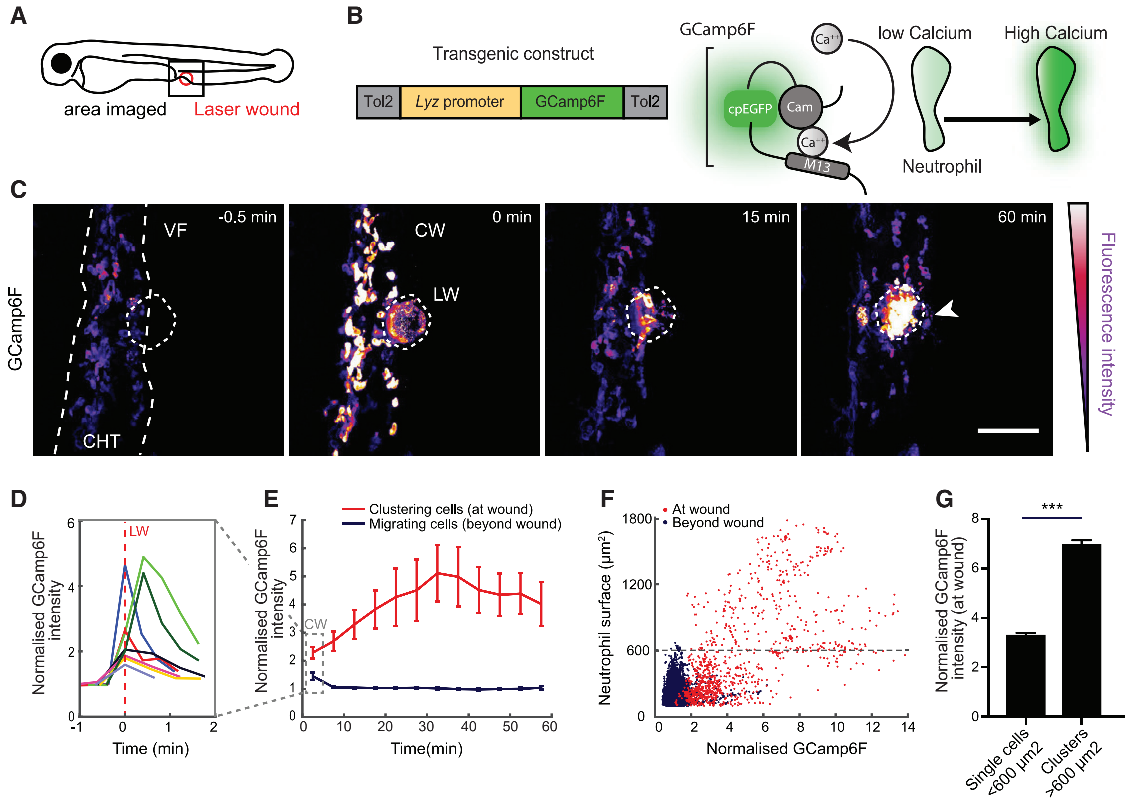

Figure 1. Calcium Dynamics in Neutrophils during Swarming

(A) Schematic of a 3-day-post-fertilization (dpf) zebrafish larva showing the area of two-photon laser wound damage and imaging.

(B) Construct expressing GCamp6F under the control of the lysozyme C promoter (*lyz*). Ca^{2+} binding to the calmodulin (Cam) domain of GCamp6F increases EGFP fluorescence.

(C) Time-lapse sequence of two-photon confocal image projections showing neutrophils (color-coded for GCamp6F intensity) migrating from the caudal hematopoietic tissue (CHT) toward a laser wound (LW) (dotted line) at the ventral fin-CHT boundary (VF/CHT) in a Tg(*lyz*:GCamp6F) larva. The calcium wave (CW) is indicated in the second panel and the neutrophil cluster with a white arrow. Scale bar represents 50 μm.

(D) Quantification of mean GCamp6F intensity over time in all neutrophils in the field of view in the first 2 min post-wound. Intensity values were normalized to the mean intensity of segmented neutrophils prior to wound. Each line represents mean intensity per experimental larva.

(E) Quantification of GCamp6F intensity over time in neutrophils clustering at the wound versus migrating neutrophils beyond the wound (dotted line in C). Intensity values were normalized as in (D). n = 8 larvae in 8 experiments. Dotted square denotes the time-bin corresponding to analysis in (D).

(F) Normalized GCamp6F intensity in relation to the surface area of segmented neutrophils. Individual dots represent single neutrophils or clustered neutrophils at the wound (red) or migrating cells beyond the wound (blue). The maximum surface area for single neutrophils is indicated with dotted line. Data are from 8 larvae in 8 experiments.

(G) Comparison of GCamp6F intensity in single (<600 μm^2) versus clustering neutrophils (>600 μm^2). n = 924 single cells and n = 347 clustered cells from 8 larvae in 8 experiments; Mann-Whitney test.

In all panels, error bars represent SEM. ***p < 0.0002. See also Figure S1 and Videos S1 and S2.

arachidonic acid into LTA4 [20]. LTA4 can be further processed to different metabolites, but neutrophils are geared to produce LTB4 [9, 24]. Thus, 5-LO peri-nuclear translocation provides a microscopically tractable readout to identify neutrophils with active LTA4/LTB4 biosynthesis (Figure 2A). To link 5-LO translocation with calcium signals, we generated a zebrafish line expressing fluorescently tagged 5-LO in neutrophils Tg(*lyz*:tRFP-5LO) and crossed this with Tg(*lyz:*GCamp6F) fish (Figure 2B). The distribution of 5-LO was constitutively nuclear, as indicated by co-localization with nuclear DAPI staining (Figures S2A–S2D). To improve resolution for these subcellular dynamics, we used spinning-disk microscopy following acute laser wounding by two-photon microscopy. We detected 5-LO translocation events in neutrophils within clusters (Figures 2C and 2E; Video S2). Though many of these events were likely obscured by overlapping cells within the cluster, the events that could be discerned were limited to a median distance of 20 μm from the wound center (Figures 2C and 2E). These translocating cells were also characterized by a markedly higher calcium level compared with non-translocating cells (Figures 2C and 2E; Video S2). As further evidence, we performed mechanical wounding in the ventral fin, as the less-compact clusters in this model facilitated the detection of 5-LO dynamics. We discovered the same trend, in that peri-nuclear 5-LO translocations were detected preferably among the clustering, calcium-fluxing cells, with a median distance of 5 μm from the wound (Figures 2D and 2F; Video S2). These data suggested that the specific calcium fluxes observed in clustering cells are associated with activation of LTB4 synthesis and

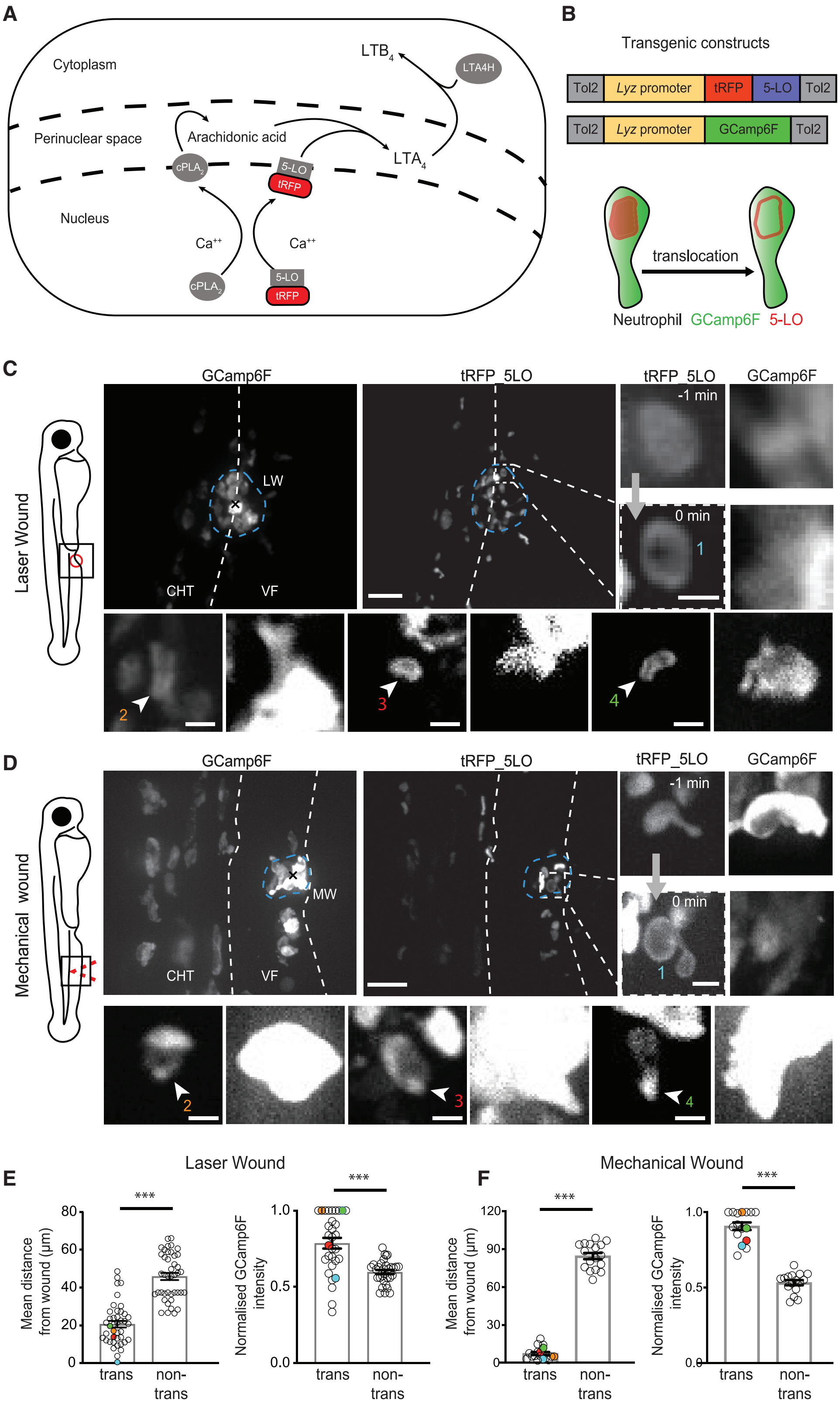

Figure 2. Activation of LTB4 Biosynthesis Is Favored in Clustering Neutrophils

(A) Schematic of LTB4 biosynthesis. cPLA2 (calcium-dependent phospholipase A2) and 5-LO are recruited to the nuclear membrane and produce arachidonic acid (AA) and LTA4, respectively. LTA4 is metabolized into LTB4 by LTA4 hydrolase.

(legend continued on next page)

that chemoattractant production is spatially favored in this cell group.

Neutrophil Calcium Fluxes Are Triggered upon Contact with Necrotic Tissue or Pioneer Fluxing Neutrophils

Based on these observations, we set out to investigate the mechanism driving the 5-LO-associated calcium fluxes in clustering neutrophils. One possibility was that the calcium fluxes resulted from passive entry, if the fluxing neutrophils were themselves in the process of dying and had lost membrane integrity. Alternatively, the calcium fluxes could have been due to active calcium entry. To explore these scenarios, we imaged neutrophil wound responses in Tg(*lyz:*GCamp6F) in the continuous presence of propidium iodide (PI) in the bath of the larva, which selectively stains nucleic acids in cells with impaired membrane integrity. The dye cannot penetrate the skin, but superficial wounding permitted transient interstitial access and local staining (Figures 3A and S2E; Video S3). Dying neutrophils were distinguished by loss of GCamp6F signal followed by uptake of PI stain, around 20–60 min post-wounding (Figures S2F and S2G; Video S3). Interestingly, the dying neutrophils would typically eject themselves outside the cluster rather than disintegrating within the cluster (Video S3). We found that the percentage of these dying/apoptotic neutrophils within the cluster was relatively low (Figure S2H) compared with the number of cells fluxing calcium. This suggested that the trigger of the 5-LO-associated calcium signals was unlikely to be limited to passive calcium entry through loss of membrane integrity. On the other hand, neutrophils underwent a calcium flux concomitant with abrupt deceleration of migration upon direct contact with necrotic tissue (Figures 3B and 3C; Video S3) or after contact with other clustering neutrophils at the wound core (Figure 3D; Video S2). This suggested that the calcium fluxes were associated with neutrophil sensing of necrotic cells and/or contact with pioneer activated neutrophils.

Extracellular Calcium Entry and ATP-Gated Calcium Channels Promote Calcium Alarm Signals

We then investigated which signaling pathways might underpin the 5-LO-associated calcium signals in neutrophils. Neutrophils can generate cytosolic calcium signals through activation of intracellular calcium stores (store-operated calcium entry [SOCE]) triggered by the inositol triphosphate (IP_3) receptor in the endoplasmic reticulum (ER) and/or plasma membrane channels, including calcium-release activated channels (CRACs), transient receptor potential (TRP) channels, and ligand-gated calcium channels (such as the ATP-gated P2X channels) [21, 25–27]. To assess the contribution of these pathways, we applied corresponding inhibitors in the bath of zebrafish larvae prior to laser wounding (Video S4). We used SKF96365, which blocks various plasma membrane calcium channels, including TRP, voltage-gated, ion-gated calcium channels, and CRACs [21]. We also used 2-APB, which blocks intracellular calcium release via the IP_3 receptor [28]. SKF96365 inhibited neutrophil intracluster calcium fluxes and the radial speed of migrating cells (Figures S3A–S3C), similar as an inhibitor of LTB4 signaling (U-75302; Figures S3A, S3E, and S3F; Video S4) [29]. Neutrophil motility levels were higher in the presence of SKF96365 and U-75302 (Figures S3D and S3G), indicating a specific defect in the overall directionality and coordination of movement rather than a defect in locomotion. By contrast, the 2-APB inhibitor did not significantly affect intracluster calcium fluxes (Figure S3E; Video S4) but affected motility, as indicated by a significant decrease of cell speed regardless of direction (Figures S3F and S3G). This suggested that plasma membrane channels are involved in the intracluster 5-LO-associated calcium fluxes.

To establish whether extracellular calcium entry is sufficient to trigger 5-LO translocation and neutrophil arrest, we added a calcium ionophore (A23187) after neutrophils initiated migration to the wound. For these experiments, we utilized the mechanical wound model to facilitate quantification of 5-LO dynamics. Within minutes after ionophore addition, neutrophils pre-clustering at the wound experienced an increase in amplitude of calcium fluxes, and this was followed by a broader formation of calcium fluxes in migrating cells further from the wound (Figures 4A and 4B; Video S4). The calcium fluxes correlated with a generic increase of 5-LO translocation across the population (Figure 4C; Video S4). Interestingly, the initial enhancement of calcium fluxes in the cells pre-clustering at the wound coincided with triggering of migration by neutrophils further away, before these latter cells were triggered to experience a calcium flux and arrest themselves (Figures 4B and 4D; Video S4). This suggested that intracluster calcium fluxes may be sufficient to trigger recruitment of nearby neutrophils, consistent with their ability to activate chemoattractant biosynthesis.

To investigate whether extracellular calcium is required for intracluster calcium signals, we added a calcium chelator in the medium (EGTA) shortly after neutrophils started accumulating at the wound. This disrupted calcium dynamics both in clustering cells and in migrating cells (Figures S3H and S3I; Video S4). Speed of motion was compromised in the migrating cells upon

(B) Constructs for transgenic expression of a fluorescent fusion of 5-LO with tRFP in neutrophils (below). Schematic of neutrophil with 5-LO nuclear translocation is shown.

(C and D) Spinning-disk confocal projections of neutrophils in 3-dpf double-transgenic Tg(*lyz*:GCamp6F)xTg(*lyz*:tRFP-5LO) zebrafish larvae after two-photon LW in the ventral fin-CHT boundary (C) or mechanical wound (MW) in the ventral fin (D). Blue dotted lines indicate the wound area occupied by clustering neutrophils. Zoomed images of examples of neutrophils with 5-LO translocation are shown. Time in relation to translocation is indicated in minutes in the first example. Examples are from three (LW) or two (MW) different larvae. Scale bars represent 50 μm and 5 μm, respectively.

(E and F) Quantification of mean distance from the wound center (x; left) and normalized GCamp6F fluorescence intensity (right) for 5-LO-translocating cells versus non-translocating cells in laser wounds (E) and mechanical fin wounds (F). GCamp6F fluorescence intensity was normalized to the most fluorescent cell in the corresponding frame.

(E) n = 41 cells (for translocating cells, each dot is a cell; for non-translocating cells, each dot represents the mean of all non-translocating cells in the same field of view; left) and n = 31 cells from 8 larvae in 5 different experiments (right).

(F) n = 17 (left) and n = 16 (right) cells from 5 larvae in 3 different experiments. Colored dots represent examples shown in individual images in (D).

Wilcoxon matched-pairs signed rank test. Error bar represents 95% confidence intervals of medians. ***$p < 0.0002$. See also Figure S2 and Video S2.

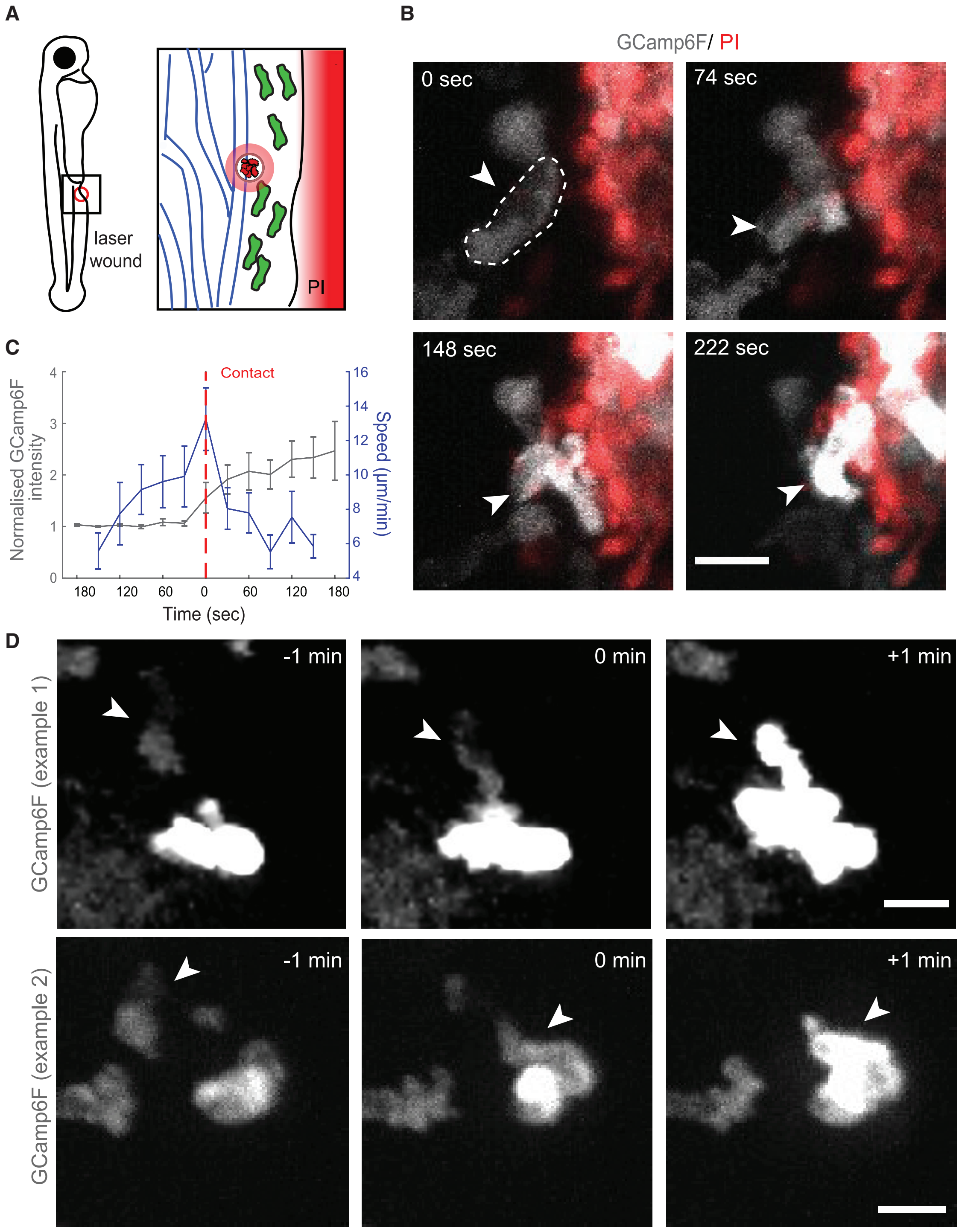

Figure 3. 5-LO-Associated Calcium Fluxes Are Triggered upon Contact with Necrotic Cells or Neutrophils with Ongoing Fluxes

(A) Schematic of two-photon laser wounding in the presence of propidium iodide (PI).

(B) Time-lapse, two-photon confocal projection images of a GCamp6F-expressing (white) neutrophil (indicated with an arrow) entering a contact with PI^+ cells/tissue (red) in a Tg(*lyz*:GCamp6F) larva; time in relation to the first frame is indicated in seconds. Scale bar represents 10 μm.

(C) Quantification of speed (blue) and normalized GCamp6F (gray) in neutrophils before and after contact with PI^+ tissue. GCamp6F intensity was normalized as in Figures 1D and 1E. Dotted red line indicates time of contact. Pooled cell data from n = 23 cells in 7 larvae and 4 experiments are shown.

(D) Examples of cell contacts transmitting calcium fluxes. Each case is represented by time-lapse images of a non-calcium-fluxing neutrophil (arrow) contacting a fluxing neutrophil. Time in minutes is indicated relative to cell-cell contact. Scale bar represents 10 μm. The quantification of neutrophil transmission of calcium fluxes is indicated in Figure 6.

Error bars represent SEM. See also Figure S2 and Video S3.

CellPress
OPEN ACCESS

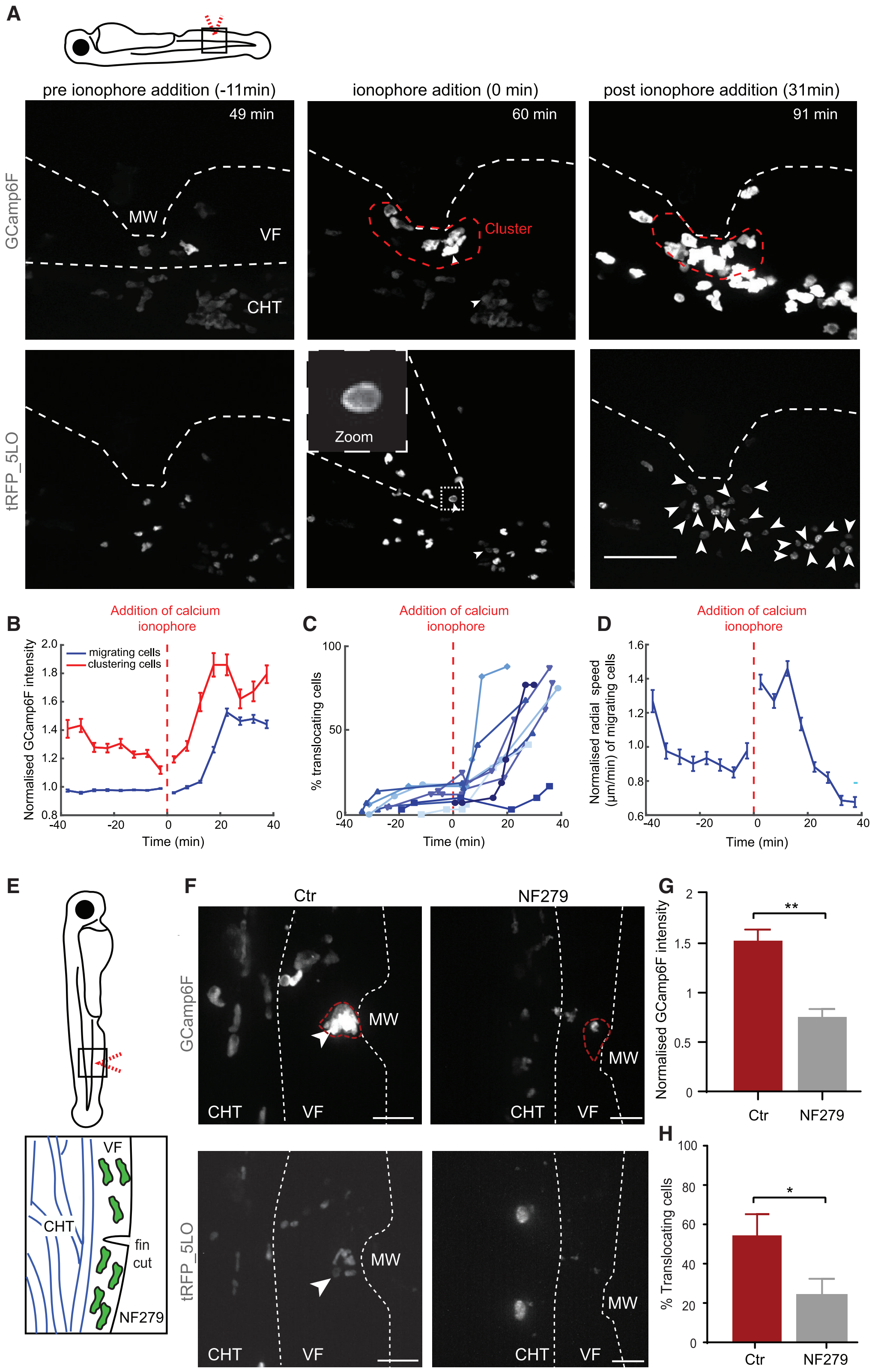

(legend on next page)

depletion of calcium (Figure S3J; Video S4). This suggested that extracellular calcium is required for intracluster calcium signals but also for motility.

Next, we tested the contribution of plasma membrane channels more specific to damage sensing, such as the inhibitor for P2X1, an ATP-gated calcium channel that has been implicated in human neutrophil calcium fluxes [27]. We found that this inhibitor (NF279) markedly suppressed calcium fluxes and 5-LO perinuclear translocation in clustering neutrophils (Figures 4E–4H; Video S4). This suggested that the prominent calcium fluxes of clustering neutrophils at the wound core act as an alarm system that depends on exposure to the damage signal ATP (we hereafter refer to this as "calcium alarm signals").

We then tested whether injection of individual chemoattractants, such as ATP, Cxcl8a, and LTB4, would be sufficient to trigger calcium alarm signals *in vivo*. We monitored neutrophil behavior and calcium dynamics 45 min after injection of these attractants in the otic cavity, an anatomical location devoid of neutrophils (Figures S4A–S4C; Video S5). None of these injections was sufficient to trigger sustained calcium alarm signals. We further investigated whether a cellular source of attractant would be effective in triggering such calcium fluxes. We monitored neutrophil behavior and calcium signals in the presence of Cxcl8a-mCherry-secreting transplanted cells, which we have previously shown to form extracellular chemokine gradients *in vivo* [30]. Neutrophils accumulated in the transplant but did not generate calcium alarm signals (Figures S4D and S4E; Video S5). This suggested that individual chemical signals, at least in the given configuration and doses, were insufficient to recapitulate the calcium fluxes seen at wounds.

Neutrophil Cx43 Is Required for Coordinated Calcium Fluxes and Swarm Initiation

We had so far identified ATP sensing as a key damage cue that triggers calcium alarm signals in swarming neutrophils. However, this did not explain the highly efficient and coordinated spread of the calcium fluxes within the clusters. We hypothesized that clustering neutrophils may be mutually reinforcing ATP signaling. Neutrophils release ATP through connexin hemichannels, but whether this affects propagation of damage sensing has not been explored [31, 32]. To test this *in vivo*, we visualized neutrophil behavior in the presence of carbenoxolone (CBX), a drug that inhibits connexin channel activity [33]. This treatment profoundly inhibited neutrophil calcium alarm signals both in laser wounds (Figures 5A and 5B; Video S6) and mechanical fin wounds (Figures S4F and S4G; Video S6). Analysis of 5-LO events in the less-compact clusters of the latter model showed a reduced probability of 5-LO translocation after CBX treatment (Figures S4F and S4H). Further quantifications in our main laser wound model showed that CBX treatment led to exploratory single-cell motility, as indicated by high motility levels but low radial speed in comparison with untreated embryos (Figures 5C and S5A).

To genetically corroborate these findings, we investigated connexin expression in purified zebrafish neutrophils and found two connexin genes to be expressed, *cx43* and *cx43.4* (Figure S5B). To assess *cx43* and *cx43.4* expression in neutrophils *in situ*, we performed whole-mount immunohistochemistry, using an antibody targeting both isoforms (Figures S5C and S5D). Cx43/Cx43.4 could be detected as vesicular puncta, usually in proximity to the membrane, in neutrophils of both wounded and unwounded larvae. Cx43/Cx43.4 was also detectable at the cell surface between contacting cells within clusters at the wound (Figures S5C and S5D). To inhibit *cx43/cx43.4* expression, we tested combinatorial CRISPR-Cas9 knockout of *cx43* and *cx43.4*. However, this led to zebrafish embryonic lethality before the onset of neutrophil development, consistent with the lethality of *cx43*-null mutations observed in mice [34]. We thus used knockdown with *cx43* morpholinos that phenocopy hypomorphic mutations of *cx43* [35]. The morpholino mixture for *cx43/cx43.4* resulted in reduced retina size, an expected developmental phenotype [35] (Figures S5E and S5F). Consistent with the CBX results, *cx43/cx43.4* knockdown reduced calcium fluxes in clustering neutrophils (Figures 5A and 5B; Video S6). *cx43/cx43.4* knockdown also compromised swarming, as indicated by reduced radial speed but increased motion levels (Figures 5C and S5A; Video S6). To assess generic chemotaxis defects, we examined whether Cx43 inhibition compromises responsiveness to chemoattractants. We found that neutrophils in Cx43 morphants showed defective neutrophil recruitment to Cxcl8a injected in the otic cavity, but not to ATP and LTB4 (Figures S6A–S6D). This suggested specific cross-talks between Cx43/Cxcl8a signaling rather than generic defects in chemotaxis.

Figure 4. ATP-Gated Calcium Channels and Extracellular Calcium Entry Promote 5-LO-Capacitating Calcium Fluxes in Neutrophils *In Vivo*

(A) Spinning-disk confocal projections from neutrophils in Tg(*lyz*:GCamp6F)xTg(*lyz*:tRFP-5LO) larvae responding to MW, before and after addition of 50 μM calcium ionophore (A23187). Time post-wounding is indicated inside the images. Arrows indicate translocation events. Red dotted line indicates area occupied by clustering neutrophils. Scale bar represents 50 μm. Cartoon indicates area of wounding.

(B) Normalized GCamp6F intensity over time in clustering versus migrating cells (cells within or beyond denoted red line in A). The time in relation to calcium ionophore addition is shown. n = 768–2,140 cells per bin (migrating) and n = 116–461 cells per bin (clustering) from 8 larvae in 3 experiments.

(C) Percentage of 5-LO-translocating neutrophils out of all neutrophils visible in the field of view. Each line represents an individual larva; 8 larvae from 3 different experiments.

(D) Normalized radial speed over time for migrating cells. n = 743–1,851 cells per bin from 8 larvae in 3 experiments.

(E) Schematic of mechanical ventral fin wounding in the presence of NF279. Blue indicates vessels of the caudal vein plexus within the caudal hematopoietic site.

(F) Spinning-disk confocal projection images of neutrophils in Tg(*lyz*:GCamp6F)xTg(*lyz*:tRFP-5LO) larvae 120 min after MW in the presence (right) or absence (left) of 10 μM NF279. Red dotted line indicates area occupied by clustering neutrophils. Scale bar represents 25 μm.

(G) Mean normalized GCamp6F intensity larvae treated or not with NF279. GCamp6F intensity was normalized as in Figure 1D. n = 9 control larvae and n = 3 NF279-treated larvae from 3 and 2 experiments, respectively; Mann-Whitney test.

(H) Percentage of translocating neutrophils out of all neutrophils recruited into the fin over 2 h. n = 7 control and n = 3 NF279-treated larvae from 3 and 2 experiments, respectively; Mann-Whitney test.

Error bars represent SEM. *p < 0.03, **p < 0.002. See also Figures S3 and S4 and Videos S4 and S5.

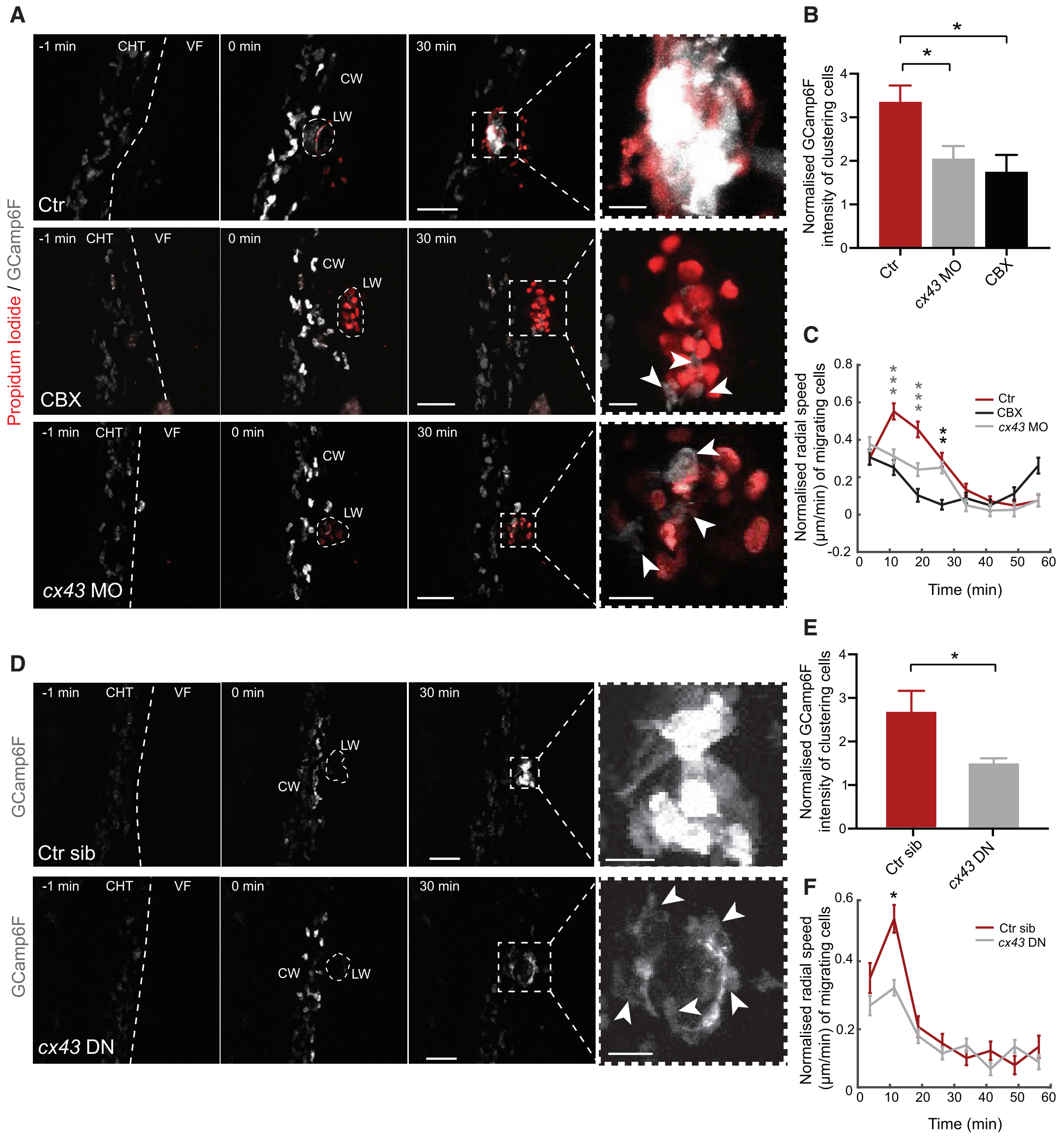

Figure 5. Neutrophil Cx43 Is Required for Intracluster Calcium Fluxes and Swarm Initiation

(A) Time-lapse sequence of two-photon confocal image projections showing neutrophils in Tg(*lyz*:GCamp6F) larvae in the presence of PI, without treatment (ctr), with 50 μM CBX, or with morpholinos against *cx43/cx43.4* (*cx43* MO). Scale bars represent 50 μm and 10 μm for zoomed-out and zoomed-in images, respectively. Time after LW is shown in minutes. Arrows indicate neutrophils in contact with the wound.

(B) Normalized GCamp6F levels in control (n = 8), CBX-treated larvae (n = 5), and *cx43* MO-treated (n = 7) from 8, 2, and 3 experiments, respectively. One-way ANOVA with Dunnett's post test is shown. GCamp6F intensity was normalized as in Figure 1D. Data are from Tg(*lyz*:GCamp6F) and Tg(*lyz*:GCamp6F)xTg(*lyz*:tRFP-5LO) larvae evenly distributed across the groups.

(C) Neutrophil radial speed over time post-wounding for neutrophils in control, *cx43* MO-treated, and CBX-treated larvae. Data are pooled from Tg(*lyz*:GCamp6F), Tg(*lyz*:GCamp6F)xTg(*lyz*:tRFP-5LO), and Tg(*mpx*:GFP)^{i114} zebrafish larvae. n = 1,201–1,719 cell steps per bin from 12 control, n = 1,268–1,535 cell steps per bin from 8 CBX-treated, and n = 1,308–1,554 cell steps per bin from 11 *cx43* MO-injected larvae from 8, 3, and 4 experiments, respectively. Kruskal-Wallis with Dunn's post test results indicating significance found between ctr and MO and between ctr and CBX (gray) or only between ctr and CBX (black) are shown.

(D) Time-lapse sequence of two-photon confocal image projections showing neutrophils in Tg(*lyz*:GCamp6F)xTg(*lyz*:*cx43DN*-T2A-mCherry) zebrafish larvae, positive (*cx43* DN) or negative for the Cx43 DN-T2A-mCherry transgene (control siblings: Ctr sib). Annotations are as in (A).

(E) Normalized GCamp6F levels in neutrophils positive (*cx43* DN) or negative for the Cx43DN-T2A-mCherry transgene (Ctr sib). n = 7 *cx43* DN transgenics and n = 5 control siblings from 3 experiments; Mann-Whitney test.

(F) Neutrophil radial speed over time post-wounding for neutrophils positive (*cx43* DN) or negative for the Cx43DN-T2A-mCherry transgene (Ctr sib). n = 1,330–1,602 cell steps per bin from 5 control siblings and n = 2,573–3,050 cell steps per bin from 7 *Cx43* DN larvae from 3 experiments; Mann-Whitney test.

Error bars represent SEM. *p < 0.03, **p < 0.002, ***p < 0.0002. See also Figures S4–S6 and Video S6.

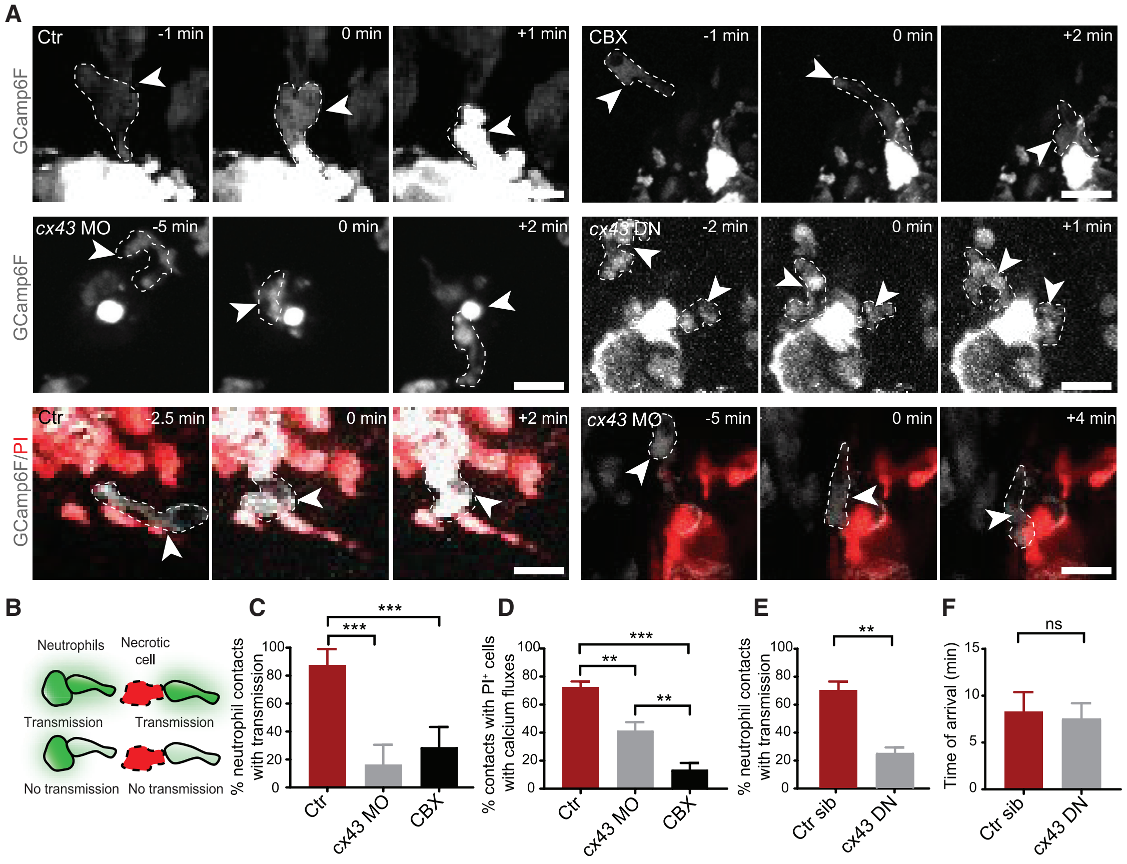

Figure 6. Neutrophil Cx43 Is Required for Autonomous and Cooperative Neutrophil Calcium Fluxes

(A) Time-lapse images showing calcium fluxes in neutrophils (arrow) contacting other neutrophils or necrotic cells in control untreated, CBX-treated, or *cx43* MO-treated Tg(*lyz*:GCamp6F) larvae or in Tg(*lyz*:GCamp6F)xTg(*lyz*:*cx43DN*-T2A-mCherry) larvae. Time in minutes is indicated relative to the start of the cell-cell contact. Scale bar represents 15 μm.

(B) Cartoon illustrates contacts between neutrophils and necrotic PI$^+$ cells or between fluxing and non-fluxing neutrophils resulting or not in calcium flux transmission.

(C and D) Percentage of neutrophil-neutrophil contacts (C) or neutrophil-PI$^+$ cell contacts (D) resulting in transmission of calcium fluxes. Data are from Tg(*lyz*:GCamp6F)xTg(*lyz*:tRFP-5LO) (C and D) or Tg(*lyz*:GCamp6F) (D) larvae. Contacts in which none of the cells is initially fluxing are not included. n = 8 control, 4 CBX-treated larvae, and 7 *Cx43* morphants from 8, 2, and 3 experiments, respectively (C). n = 5 control, 5 CBX-treated larvae, and 5 *Cx43* morphants from 5, 2, and 2 experiments, respectively (D). One-way ANOVA, Tukey's multiple comparisons test, is shown.

(E) Percentage of contacts resulting in transmission of calcium fluxes in Tg(*lyz*:GCamp6F)xTg(*lyz*:*cx43DN*-T2A-mCherry) zebrafish larvae, positive (*cx43* DN) or negative for the Cx43DN-T2A-mCherry transgene (control siblings: Ctr sib). n = 7 *cx43* DN and n = 5 control siblings from 3 experiments; Mann-Whitney test.

(F) Time point of arrival of first neutrophil at the wound in neutrophils positive (*cx43* DN) or negative for the Cx43DN-T2A-mCherry transgene (control siblings: Ctr sib). n = 7 *cx43* DN and n = 5 control siblings from 3 experiments; Mann Whitney test.

Error bars represent SEM. **p < 0.002, ***p < 0.0002. See also Video S6.

Given the broad expression of *cx43*, we next interrogated whether neutrophil *cx43* is important for neutrophil swarming. To this end, we generated transgenic zebrafish whereby neutrophils express a dominant-negative version of *cx43* (Tg(*lyz:cx43DN*-T2A-mCherry)), which inhibits Cx43 channel activity [36]. The behavior of neutrophils in these transgenics was similar as in *cx43* morphants, in that they showed reduced whole-cell calcium fluxes and less-coordinated motility (Figures 5D–5F; Video S6). Assessment of neutrophil accumulation at fixed time points across a large pool of embryos showed that inhibition of neutrophil Cx43 suppressed neutrophil accumulation to a similar degree as global Cx43 inhibition (Figures S6E and S6F). This suggested that neutrophil Cx43 largely accounts for the overall defect in neutrophil accumulation at wounds. The accumulation defects were not due to suppressed motility, as neutrophil speed was higher in Cx43 DN mutants versus siblings (Figure S6G). Altogether, this evidence demonstrated an important role for neutrophil Cx43/Cx43.4 in coordinating intracluster calcium signaling and swarming.

Cx43 subunits can assemble into hemichannels that allow passage of ions and small molecules (including ATP) to/from the extracellular environment or into gap junctions that allow such conductivity across cells [37]. It remained unclear whether Cx43 mediates gap-junctional coupling or hemichannel-based ATP release and signaling [32, 38, 39] in clustering neutrophils. We did not observe PI uptake in live neutrophils, suggesting either absence of hemichannel activity or that the level of

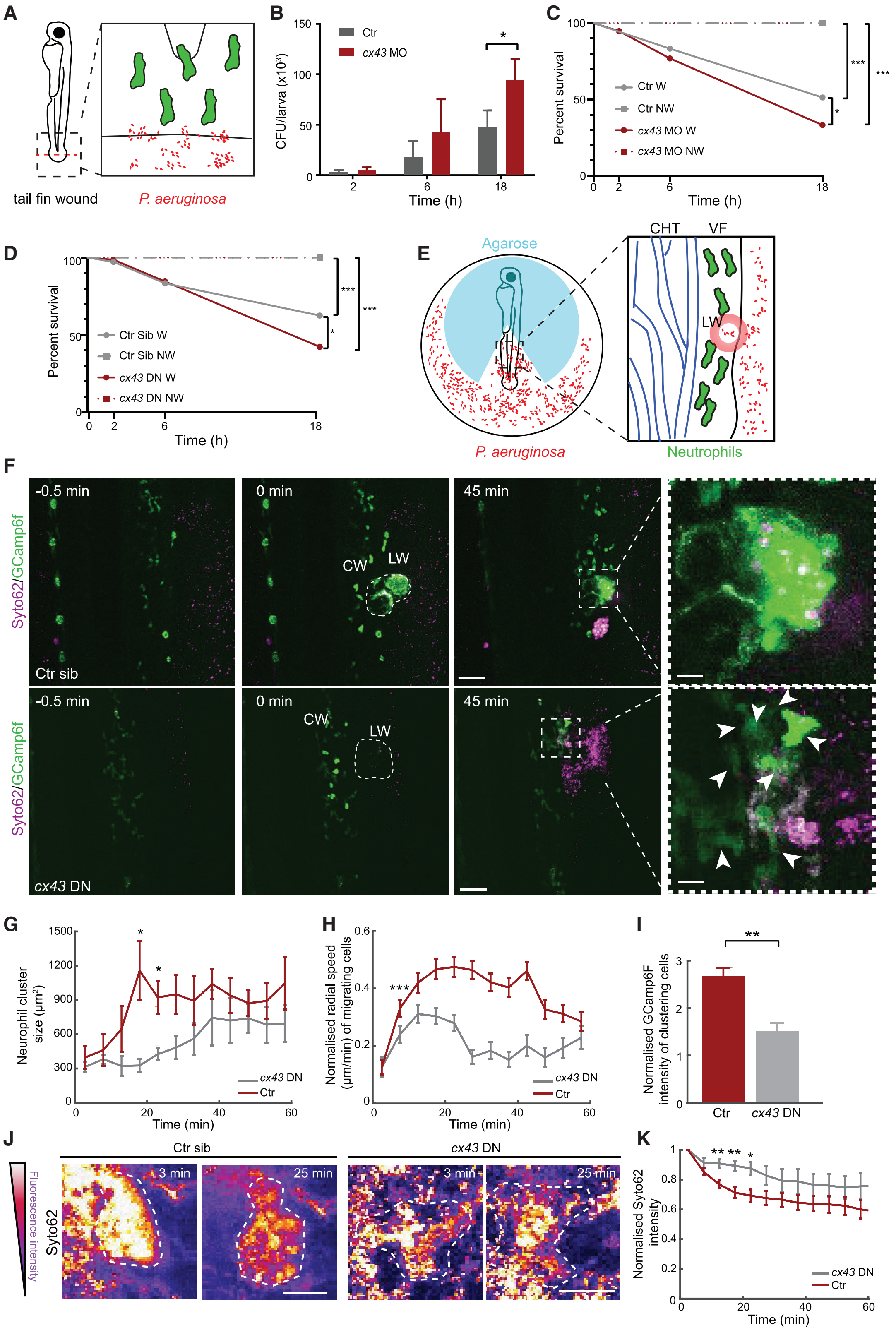

(legend on next page)

transport is below our detection limit (hemichannel opening could be transient and brief, unlike the permanent membrane integrity disruption in necrotic cells). We thus used functional tests to interrogate a link between Cx43 and ATP signaling in neutrophil swarming *in vivo*. Specifically, we found that Cx43 inhibition did not cause further reduction in neutrophil accumulation in NF279-treated larvae (Figures S6H and S6I). The absence of additive effects suggested that Cx43 and P2X1 may act in the same pathway, which would be consistent with hemichannel function of Cx43 in releasing ATP.

To better understand the role of Cx43, we quantified separately the pioneer calcium fluxes upon contact with necrotic tissue versus the subsequent transmission of these fluxes to other neutrophils. All types of Cx43 inhibition (CBX, *cx43/cx43.4* knockdown, and expression of *cx43 DN*) reduced neutrophil calcium fluxes both upon contact with necrotic tissue and upon contacting other fluxing neutrophils (Figures 6A–6E; Video S6). This suggested that Cx43 likely plays a role in both autonomous and cooperative amplification of neutrophil damage signaling in the clusters. On the other hand, Cx43 inhibition did not affect the initial recruitment of pioneer neutrophils, as these arrived at the wound within the same time frame (Figure 6F).

Cx43 Promotes Wound Defense from Bacterial Invasion

In sterile injury, neutrophil swarms appear detrimental to tissue integrity, as they cause local tissue disruption [8, 9]. In certain parasitic infections, neutrophils promote pathogen spreading [40], and swarms could, in principle, facilitate this. The possible evolutionary benefit of neutrophil swarming is unclear, given these pathological implications. We hypothesized that dense neutrophil clusters, as opposed to diffuse patrolling, might provide a particularly effective antimicrobial barrier in breached tissue exposed to free-living opportunistic pathogens. To this end, we established a relevant wound infection model in zebrafish. *Pseudomonas aeruginosa* is an opportunistic bacterial pathogen that causes nosocomial wound infections [41, 42]. Wounds are nutrient-rich environments for these bacteria, which actively colonize these niches through chemotaxis [42, 43]. Acute wound infections by *P. aeruginosa* often spread rapidly, leading to sepsis and mortality within days or weeks [44]. We examined whether this is recapitulated in zebrafish by incubating larvae that had been mechanically wounded in a medium with PAO1 *P. aeruginosa* (Figure 7A). Non-wounded larvae were resistant to infection across a range of bacterial infection doses (Figure S7). In contrast, wounded larvae showed a dose-dependent decrease in survival (Figure S7), which correlated with an increase in total whole-body bacterial burden (Figures 7B and 7C). Using an intermediate infection dose, we found that *cx43* morphant larvae showed significantly reduced survival and increased bacterial burden as early as 18 h after infection (Figures 7B and 7C). We obtained similar results by comparing infection-induced morbidity in *lyz:cx43DN*-T2A-mCherry larvae and control siblings (Figure 7D). These results demonstrated that Cx43 plays a role in restricting wound infections by pathogenic bacteria.

To link these findings with neutrophil swarm defects, we developed live imaging of wound infection by fluorescently labeled PAO1 *P. aeruginosa* (Figure 7E). Strikingly, wounding was followed by a sharp flow of bacteria toward the wound within 5 min post-wounding (Video S7). This was accompanied by neutrophil swarming with maximal clustering and cell coordination within 20 min (Figures 7F–7H), thus with comparable initiation kinetics as in sterile wounds. The neutrophil clusters were characterized by similar intracluster calcium signals as in sterile wounds (Figure 7I). To determine the function of clusters in bacterial fate, we analyzed the burden of bacteria in the area occupied by the neutrophil cluster (Video S7). We observed a rapid clearance of bacteria upon neutrophil clustering, as indicated by a decay in fluorescent signal (Figures 7J and 7K). Neutrophils expressing *cx43* DN showed a delay in bacterial clearance (Figures 7J and 7K), consistent with defects in calcium alarm signals and neutrophil swarming in this time frame (Figures 7F–7I). Thus, the contribution of neutrophil Cx43 in wound immunity could at least in part be explained by its role in neutrophil clustering in this breached locus.

Figure 7. Cx43 Is Required for Maximal Wound Defense from Bacterial Invasion

(A) Schematic of tail amputation and infection by PAO1 *P. aeruginosa*.

(B) Colony-forming units (CFUs) per larva in control wild-type (AB strain), non-injected larvae or *cx43* MO-injected larvae. Time after wounding is in hours (h). n = 4 experiments, with 5 larvae per group; Mann-Whitney test.

(C) Survival over time in control wild-type, non-injected larvae or *cx43* MO-injected larvae, wounded (W) or not (NW) in the presence of PAO1. n = 4 experiments, with 20 larvae per group; log rank (Mantel-Cox) test.

(D) Survival over time in Tg(*lyz*:GCamp6F)xTg(*lyz*:*cx43DN*-T2A-mCherry) zebrafish larvae, positive (*cx43* DN) or negative for the Cx43DN-T2A-mCherry transgene (control siblings: Ctr sib), wounded (W) or not (NW) in the presence of PAO1. n = 4 experiments, with 20 larvae per group; log rank (Mantel-Cox) test.

(E) Schematic of imaging wound infection. Annotations are as in Figure 4E.

(F) Time-lapse sequence of two-photon confocal projections showing neutrophils in zebrafish larvae, positive (*cx43* DN) or negative for the Cx43DN-T2A-mCherry transgene (control siblings; Ctr sib), in the presence of Syto62-labeled PAO1. Scale bars represent 50 μm and 10 μm. CW, CHT, and VF are as in Figure 1C. Arrows indicate neutrophils in contact with the wound. Dotted lines outline neutrophil clusters.

(G) Neutrophil cluster size over time post-wounding in *cx43* DN or control larvae (Ctr) (includes negative siblings and single Tg(*lyz*:GCamp6F) transgenics). n = 5 *cx43* DN and 7 control larvae from 6 and 5 experiments, respectively; Mann-Whitney test.

(H) Neutrophil radial speed over time post-wounding in *cx43* DN or control larvae. n = 1,290–1,537 cell steps per bin from 7 control larvae and n = 1,527–2,079 cell-steps per bin from 8 *cx43* DN larvae imaged in 6 and 5 experiments, respectively; Mann-Whitney test.

(I) GCamp6F levels normalized as in Figure 1D. n = 5 *cx43DN* transgenics and n = 7 control larvae from 6 and 5 experiments, respectively; Mann-Whitney test.

(J) Images of the wound (dotted outline) pseudocolored for fluorescence intensity of Syto62-labeled PAO1 bacteria. Time post-wounding is indicated in minutes. Scale bar represents 25 μm.

(K) Fluorescence intensity of bacteria at the wound relative to maximal initial intensity in this area. n = 5 *Cx43* DN and n = 8 control larvae from 6 and 5 experiments, respectively; Mann-Whitney test.

Error bars represent SEM. *p < 0.03, **p < 0.002, ***p < 0.0002. See also Figure S7 and Video S7.

DISCUSSION

Neutrophil accumulation in inflamed tissue has pervasive implications in disease, and therapeutic strategies to fine-tune this process are desirable. A better understanding of how this migratory response physiologically escalates is important in this endeavor. Here, we reveal a cascade of signaling events underpinning amplification of neutrophil migration into prominent swarms. Our experiments reveal that Cx43 hemichannels drive coordinated calcium fluxes in a nascent neutrophil cluster, which promotes swarm growth by locally enhancing activation of chemoattractant biosynthesis. As Cx43 hemichannels mediate ATP release [27, 32], we propose that neutrophil Cx43 hemichannels amplify damage sensing in an autocrine and juxtacrine fashion at the wound focus to assemble a centralized, powerful chemoattractant gradient source. We show that formation of dense clusters through this mechanism is beneficial for early protection of the wound margin from opportunistic bacterial invasion.

Our study fills an important gap in our understanding of neutrophil swarming, as the basis of cell coordination has so far remained unclear. Our model provides a plausible explanation for why neutrophil access to the necrotic site is crucial for initiation of swarming [14] and why a primary neutrophil cluster precedes the onset of rapid aggregation [8, 15, 45]. The model also has implications in how an effective LTB4 gradient is self-generated by neutrophils during swarming [8]. The theoretical range of a gradient is dependent on the concentration of signal produced at the source and its diffusion and degradation rate [46]. The cooperative reinforcement of 5-LO-capacitating calcium fluxes combined with the rapid tissue diffusion of LTB4 [47] could play a part in the radius of the corresponding chemical gradient [8]. This does not exclude the contribution of LTB4-containing exosomes in the process, whose slower release and propagation could have additional effects in the response [48, 49]. Our data revealed a key role for neutrophil Cx43 in coordinating calcium alarm signals and swarm initiation. This mechanism could in part account for the reduced neutrophil accumulation in wounds of Cx43-deficient mice, a phenotype previously presumed to involve endothelial Cx43 [50].

Our wound infection data suggest an evolutionary benefit for neutrophil clustering. We found that the wound core is rapidly colonized by opportunistic bacteria within 5 min, which are largely cleared in this locus by neutrophil swarms within the following 20 min. This process was delayed when Cx43 and neutrophil swarms were inhibited. We speculate that suboptimal sealing of the wound by dense neutrophil clusters facilitates entry of bacteria into the deeper tissue. Such differences during the initial wound invasion could have knockon effects on subsequent host-pathogen interactions and, at least in part, account for the increased infection-induced morbidity in Cx43-inhibited larvae. The role of Cx43 and neutrophil swarming in wound defense could be relevant to other opportunistic bacteria capable of infecting wounds [51]. This does not exclude the possibility that other types of pathogens that propagate through neutrophils, such as *Leishmania major* [40], might exploit dense swarms for dissemination.

The coordination mechanism we describe is distinct from previous paradigms of collective cell migration. In a cohesive migrating group of cells, such as neural crest cells or the lateral line primordium, intercellular adhesion is critical for coordination of motion [52]. Cohesiveness further allows self-determination of directionality through asymmetric distribution of receptors across the moving cell mass [53, 54]. In the non-cohesive paradigm of slime mold aggregation, pulsatile release of attractant underpins coordinated gathering toward a single cell [11]. Here, we show that intercellular signal amplification within a seeding cluster powers the formation of a strong attractant source. This simple mechanistic principle appears to balance the benefit of rapid escalation with the risk of excess or misdirected congregation. The requirement for close cooperation in the primary cluster provides a level of stringency in the initiation of swarming. The preferential activation of attractant biosynthesis at the wound core provides spatial precision.

Our study points to interesting future lines of inquiry. One issue that remains unclear is why calcium alarm signals are spatially restricted in the wound-occupying cluster. P2X1 channel opening requires a threshold level of ATP, and such levels might be more likely encountered at the wound core if connexin hemichannels are selectively activated in this locus. Cx43 channel opening can be activated by various stimuli *in vitro*, such as LPS, LTB4, fMLP, and changes in extracellular ions [27, 32]. We reason that pioneer neutrophils contacting necrotic tissue initially experience death-specific signals (such as fMLP) that activate Cx43 opening. Subsequent calcium fluxes, 5-LO activation, and LTB4 release could then activate secondary Cx43 channel opening in a positive feedback loop [32]. This could account for the sustained duration of intracluster calcium fluxes and the inhibition of these fluxes by the LTB4 signaling blocker (U-75302). A positive feedback loop between LTB4 and Cx43 might also explain the suppression of intracluster calcium fluxes by the SOCE inhibitor SKF96365, as the latter blocks neutrophil activation by LTB4 [55]. Mechanosensation could be an intriguing cofactor in spatially restricting calcium alarm signals, as mechanical stimulation of the nucleus contributes to 5-LO activation [56]. A requirement for coincidence of multiple signals might explain why injection of individual chemical cues, including ATP, was insufficient to trigger prominent intracluster calcium fluxes in neutrophils. Another question raised by our study is whether centralized attractant production is sufficient to generate swarms and how many neutrophils might be required to form an effective gradient source. This would be interesting to address through *in silico* modeling and generation of optogenetic tools to manipulate such dynamics [57, 58]. Finally, a key point to elucidate will be the factors that terminate neutrophil swarming. Although the duration of 5-LO translocation was difficult to track in the dense clusters, it is noteworthy that translocation events and calcium fluxes were detectable beyond the duration of the migration wave. As monocyte/macrophage recruitment correlates with cessation of swarms in mice [8] and promotes neutrophil wound departure in zebrafish [59], it would be interesting to explore the role of these cells in catabolism of neutrophil-derived LTA4/LTB4.

Altogether, our study describes a novel mechanistic paradigm of collective cell behavior and identifies connexin channels as a key determinant of neutrophil swarming and wound immunity. This opens several avenues for investigation of this pathway in physiological and pathological conditions.

STAR★METHODS

Detailed methods are provided in the online version of this paper and include the following:

- KEY RESOURCES TABLE
- RESOURCE AVAILABILITY
  - Lead Contact
  - Materials Availability
  - Data and Code Availability
- EXPERIMENTAL MODEL AND SUBJECT DETAILS
- METHOD DETAILS
  - DNA constructs and transgenic zebrafish lines
  - Morpholino injections
  - Western blotting
  - Two-photon laser wound and live imaging
  - Tail fin wound infection experiments
  - Two-photon imaging of wound colonization by bacteria
  - Chemoattractant injections in the otic cavity
  - Whole-mount Sudan Black staining and immunohistochemistry
  - Extraction of cell trajectories
  - Extraction of cell surface data
  - Definition of wound perimeter and of clustering versus migrating cells
  - Quantification of GCamp6F levels
  - Quantification of GCamp6F levels with neutrophil cluster size
  - Calculation of neutrophil radial speed
  - Detection and scoring of 5-LO translocation in zebrafish neutrophils
  - Analysis of 5-LO translocation in relation to distance or GCamp6F intensity
  - Analysis of GCamp6F in neutrophil cell-cell contacts and neutrophil contacts with necrotic tissue
  - Evolution of neutrophil GCamp6F levels and speed over time and upon contact with necrotic cells
  - Analysis of fluorescence intensity of bacteria over time
- QUANTIFICATION AND STATISTICAL ANALYSIS

SUPPLEMENTAL INFORMATION

Supplemental Information can be found online at https://doi.org/10.1016/j.cub.2020.05.030.

ACKNOWLEDGMENTS

We thank Philippe Bousso, Menna Clatworthy, Ewa Paluch, and Rob White for comments on the manuscript; Kevin O' Holleran for two-photon microscopy; Bill Harris and Christine Holt groups for confocal microscopy; Nachiket Kashikar for the GCamp6F cDNA; Anna Huttenlocher for the *lyz* backbone vector; J.P. Levraud for cDNA of adult zebrafish; Steve Renshaw for the Tg(*mpx*:GFP)^{i114} line; Martin Welch for provision of the *Pseudomonas aeruginosa* PAO1 strain; and Michael Redd for advice in imaging wound infections. H.P. was supported by a Wellcome Trust PhD grant (105391/Z/14/Z). M.S. and the research were supported by a Medical Research Council Career Development Award (MR/L019523/1), a Wellcome Trust (204845/Z/16/Z), Isaac Newton Trust (12.21 (a)i), and a Royal Society Research Grant (RG170247). M.B. was supported by an Erasmus programme (Master de Biologie, École Normale Supérieure de Lyon). C.C. and H.A.W. were supported by a Medical Research Council Doctoral Training Programme. F.P. was supported by an Erasmus programme (University of Athens).

AUTHOR CONTRIBUTIONS

Conceptualization, M.S.; Methodology, M.S. and H.P.; Software, A.G.; Formal Analysis, A.G. and H.P.; Data Curation, A.G. and H.P.; Visualization, H.P. and M.S.; Investigation, H.P., M.B., H.A.W., C.C., and F.P.; Writing – Original Draft, M.S. and H.P.; Writing – Review & Editing, M.S.; Funding Acquisition, M.S. and H.P.; Project Administration, M.S. and H.P.; Supervision, M.S.

DECLARATION OF INTERESTS

The authors declare no competing interests.

Received: January 31, 2020
Revised: March 30, 2020
Accepted: May 7, 2020
Published: June 4, 2020

REFERENCES

1. Kolaczkowska, E., and Kubes, P. (2013). Neutrophil recruitment and function in health and inflammation. Nat. Rev. Immunol. *13*, 159–175.
2. Wang, J. (2018). Neutrophils in tissue injury and repair. Cell Tissue Res. *371*, 531–539.
3. Singel, K.L., and Segal, B.H. (2016). Neutrophils in the tumor microenvironment: trying to heal the wound that cannot heal. Immunol. Rev. *273*, 329–343.
4. Soehnlein, O., Steffens, S., Hidalgo, A., and Weber, C. (2017). Neutrophils as protagonists and targets in chronic inflammation. Nat. Rev. Immunol. *17*, 248–261.
5. Futosi, K., Fodor, S., and Mócsai, A. (2013). Neutrophil cell surface receptors and their intracellular signal transduction pathways. Int. Immunopharmacol. *17*, 638–650.
6. Afonso, P.V., Janka-Junttila, M., Lee, Y.J., McCann, C.P., Oliver, C.M., Aamer, K.A., Losert, W., Cicerone, M.T., and Parent, C.A. (2012). LTB4 is a signal-relay molecule during neutrophil chemotaxis. Dev. Cell *22*, 1079–1091.
7. Enyedi, B., Kala, S., Nikolich-Zugich, T., and Niethammer, P. (2013). Tissue damage detection by osmotic surveillance. Nat. Cell Biol. *15*, 1123–1130.
8. Lämmermann, T., Afonso, P.V., Angermann, B.R., Wang, J.M., Kastenmüller, W., Parent, C.A., and Germain, R.N. (2013). Neutrophil swarms require LTB4 and integrins at sites of cell death in vivo. Nature *498*, 371–375.
9. Kienle, K., and Lämmermann, T. (2016). Neutrophil swarming: an essential process of the neutrophil tissue response. Immunol. Rev. *273*, 76–93.
10. McDonald, B., Pittman, K., Menezes, G.B., Hirota, S.A., Slaba, I., Waterhouse, C.C.M., Beck, P.L., Muruve, D.A., and Kubes, P. (2010). Intravascular danger signals guide neutrophils to sites of sterile inflammation. Science *330*, 362–366.
11. Dormann, D., Vasiev, B., and Weijer, C.J. (2002). Becoming multicellular by aggregation; the morphogenesis of the social amoebae Dicyostelium discoideum. J. Biol. Phys. *28*, 765–780.
12. Kriebel, P.W., Barr, V.A., Rericha, E.C., Zhang, G., and Parent, C.A. (2008). Collective cell migration requires vesicular trafficking for chemoattractant delivery at the trailing edge. J. Cell Biol. *183*, 949–961.
13. Sarris, M., and Sixt, M. (2015). Navigating in tissue mazes: chemoattractant interpretation in complex environments. Curr. Opin. Cell Biol. *36*, 93–102.
14. Uderhardt, S., Martins, A.J., Tsang, J.S., Lämmermann, T., and Germain, R.N. (2019). Resident macrophages cloak tissue microlesions to prevent neutrophil-driven inflammatory damage. Cell *177*, 541–555.e17.

15. Park, S.A., Choe, Y.H., Park, E., and Hyun, Y.-M. (2018). Real-time dynamics of neutrophil clustering in response to phototoxicity-induced cell death and tissue damage in mouse ear dermis. Cell Adhes. Migr. *12*, 424–431.
16. Harvie, E.A., and Huttenlocher, A. (2015). Neutrophils in host defense: new insights from zebrafish. J. Leukoc. Biol. *98*, 523–537.
17. Peters-Golden, M., Canetti, C., Mancuso, P., and Coffey, M.J. (2005). Leukotrienes: underappreciated mediators of innate immune responses. J. Immunol. *174*, 589–594.
18. Vincent, W.J.B., Harvie, E.A., Sauer, J.-D., and Huttenlocher, A. (2017). Neutrophil derived LTB4 induces macrophage aggregation in response to encapsulated Streptococcus iniae infection. PLoS ONE *12*, e0179574.
19. Reátegui, E., Jalali, F., Khankhel, A.H., Wong, E., Cho, H., Lee, J., Serhan, C.N., Dalli, J., Elliott, H., and Irimia, D. (2017). Microscale arrays for the profiling of start and stop signals coordinating human-neutrophil swarming. Nat. Biomed. Eng. *1*, 0094.
20. Luo, M., Jones, S.M., Peters-Golden, M., and Brock, T.G. (2003). Nuclear localization of 5-lipoxygenase as a determinant of leukotriene B4 synthetic capacity. Proc. Natl. Acad. Sci. USA *100*, 12165–12170.
21. Beerman, R.W., Matty, M.A., Au, G.G., Looger, L.L., Choudhury, K.R., Keller, P.J., and Tobin, D.M. (2015). Direct in vivo manipulation and imaging of calcium transients in neutrophils identify a critical role for leading-edge calcium flux. Cell Rep. *13*, 2107–2117.
22. Chen, T.-W., Wardill, T.J., Sun, Y., Pulver, S.R., Renninger, S.L., Baohan, A., Schreiter, E.R., Kerr, R.A., Orger, M.B., Jayaraman, V., et al. (2013). Ultrasensitive fluorescent proteins for imaging neuronal activity. Nature *499*, 295–300.
23. Razzell, W., Evans, I.R., Martin, P., and Wood, W. (2013). Calcium flashes orchestrate the wound inflammatory response through DUOX activation and hydrogen peroxide release. Curr. Biol. *23*, 424–429.
24. Serhan, C.N., and Sheppard, K.A. (1990). Lipoxin formation during human neutrophil-platelet interactions. Evidence for the transformation of leukotriene A4 by platelet 12-lipoxygenase in vitro. J. Clin. Invest. *85*, 772–780.
25. Lecut, C., Frederix, K., Johnson, D.M., Deroanne, C., Thiry, M., Faccinetto, C., Marée, R., Evans, R.J., Volders, P.G.A., Bours, V., and Oury, C. (2009). P2X1 ion channels promote neutrophil chemotaxis through Rho kinase activation. J. Immunol. *183*, 2801–2809.
26. Lindemann, O., Umlauf, D., Frank, S., Schimmelpfennig, S., Bertrand, J., Pap, T., Hanley, P.J., Fabian, A., Dietrich, A., and Schwab, A. (2013). TRPC6 regulates CXCR2-mediated chemotaxis of murine neutrophils. J. Immunol. *190*, 5496–5505.
27. Wang, X., Qin, W., Xu, X., Xiong, Y., Zhang, Y., Zhang, H., and Sun, B. (2017). Endotoxin-induced autocrine ATP signaling inhibits neutrophil chemotaxis through enhancing myosin light chain phosphorylation. Proc. Natl. Acad. Sci. USA *114*, 4483–4488.
28. Wu, H.-J., Fong, T.-H., Chen, S.-L., Wei, J.-C., Wang, I.-J., Wen, C.-C., Chang, C.-Y., Chen, X.-G., Chen, W.-Y., Chen, H.-M., et al. (2015). Perturbation of cytosolic calcium by 2-aminoethoxydiphenyl borate and caffeine affects zebrafish myofibril alignment. J. Appl. Toxicol. *35*, 287–294.
29. Tobin, D.M., Vary, J.C., Jr., Ray, J.P., Walsh, G.S., Dunstan, S.J., Bang, N.D., Hagge, D.A., Khadge, S., King, M.-C., Hawn, T.R., et al. (2010). The lta4h locus modulates susceptibility to mycobacterial infection in zebrafish and humans. Cell *140*, 717–730.
30. Sarris, M., Masson, J.-B., Maurin, D., Van der Aa, L.M., Boudinot, P., Lortat-Jacob, H., and Herbomel, P. (2012). Inflammatory chemokines direct and restrict leukocyte migration within live tissues as glycan-bound gradients. Curr. Biol. *22*, 2375–2382.
31. Bao, Y., Ledderose, C., Seier, T., Graf, A.F., Brix, B., Chong, E., and Junger, W.G. (2014). Mitochondria regulate neutrophil activation by generating ATP for autocrine purinergic signaling. J. Biol. Chem. *289*, 26794–26803.
32. Eltzschig, H.K., Eckle, T., Mager, A., Küper, N., Karcher, C., Weissmüller, T., Boengler, K., Schulz, R., Robson, S.C., and Colgan, S.P. (2006). ATP release from activated neutrophils occurs via connexin 43 and modulates adenosine-dependent endothelial cell function. Circ. Res. *99*, 1100–1108.
33. De Vuyst, E., Decrock, E., De Bock, M., Yamasaki, H., Naus, C.C., Evans, W.H., and Leybaert, L. (2007). Connexin hemichannels and gap junction channels are differentially influenced by lipopolysaccharide and basic fibroblast growth factor. Mol. Biol. Cell *18*, 34–46.
34. Lo, C.W., Waldo, K.L., and Kirby, M.L. (1999). Gap junction communication and the modulation of cardiac neural crest cells. Trends Cardiovasc. Med. *9*, 63–69.
35. Hoptak-Solga, A.D., Nielsen, S., Jain, I., Thummel, R., Hyde, D.R., and Iovine, M.K. (2008). Connexin43 (GJA1) is required in the population of dividing cells during fin regeneration. Dev. Biol. *317*, 541–548.
36. Oyamada, Y., Zhou, W., Oyamada, H., Takamatsu, T., and Oyamada, M. (2002). Dominant-negative connexin43-EGFP inhibits calcium-transient synchronization of primary neonatal rat cardiomyocytes. Exp. Cell Res. *273*, 85–94.
37. Ribeiro-Rodrigues, T.M., Martins-Marques, T., Morel, S., Kwak, B.R., and Girão, H. (2017). Role of connexin 43 in different forms of intercellular communication - gap junctions, extracellular vesicles and tunnelling nanotubes. J. Cell Sci. *130*, 3619–3630.
38. Anselmi, F., Hernandez, V.H., Crispino, G., Seydel, A., Ortolano, S., Roper, S.D., Kessaris, N., Richardson, W., Rickheit, G., Filippov, M.A., et al. (2008). ATP release through connexin hemichannels and gap junction transfer of second messengers propagate Ca2+ signals across the inner ear. Proc. Natl. Acad. Sci. USA *105*, 18770–18775.
39. Kang, J., Kang, N., Lovatt, D., Torres, A., Zhao, Z., Lin, J., and Nedergaard, M. (2008). Connexin 43 hemichannels are permeable to ATP. J. Neurosci. *28*, 4702–4711.
40. Peters, N.C., Egen, J.G., Secundino, N., Debrabant, A., Kimblin, N., Kamhawi, S., Lawyer, P., Fay, M.P., Germain, R.N., and Sacks, D. (2008). In vivo imaging reveals an essential role for neutrophils in leishmaniasis transmitted by sand flies. Science *321*, 970–974.
41. Mesaros, N., Nordmann, P., Plésiat, P., Roussel-Delvallez, M., Van Eldere, J., Glupczynski, Y., Van Laethem, Y., Jacobs, F., Lebecque, P., Malfroot, A., et al. (2007). Pseudomonas aeruginosa: resistance and therapeutic options at the turn of the new millennium. Clin. Microbiol. Infect. *13*, 560–578.
42. Turner, K.H., Everett, J., Trivedi, U., Rumbaugh, K.P., and Whiteley, M. (2014). Requirements for Pseudomonas aeruginosa acute burn and chronic surgical wound infection. PLoS Genet. *10*, e1004518.
43. Schwarzer, C., Fischer, H., and Machen, T.E. (2016). Chemotaxis and binding of Pseudomonas aeruginosa to scratch-wounded human cystic fibrosis airway epithelial cells. PLoS ONE *11*, e0150109.
44. McManus, A.T., Mason, A.D., Jr., McManus, W.F., and Pruitt, B.A., Jr. (1985). Twenty-five year review of Pseudomonas aeruginosa bacteremia in a burn center. Eur. J. Clin. Microbiol. *4*, 219–223.
45. Chtanova, T., Schaeffer, M., Han, S.-J., van Dooren, G.G., Nollmann, M., Herzmark, P., Chan, S.W., Satija, H., Camfield, K., Aaron, H., et al. (2008). Dynamics of neutrophil migration in lymph nodes during infection. Immunity *29*, 487–496.
46. Crick, F. (1970). Diffusion in embryogenesis. Nature *225*, 420–422.
47. Demy, D.L., Tauzin, M., Lancino, M., Le Cabec, V., Redd, M., Murayama, E., Maridonneau-Parini, I., Trede, N., and Herbomel, P. (2017). Trim33 is essential for macrophage and neutrophil mobilization to developmental or inflammatory cues. J. Cell Sci. *130*, 2797–2807.
48. Lim, K., Hyun, Y.-M., Lambert-Emo, K., Capece, T., Bae, S., Miller, R., Topham, D.J., and Kim, M. (2015). Neutrophil trails guide influenza-specific $CD8^+$ T cells in the airways. Science *349*, aaa4352.
49. Majumdar, R., Tavakoli Tameh, A., and Parent, C.A. (2016). Exosomes mediate LTB4 release during neutrophil chemotaxis. PLoS Biol. *14*, e1002336.
50. Qiu, C., Coutinho, P., Frank, S., Franke, S., Law, L.Y., Martin, P., Green, C.R., and Becker, D.L. (2003). Targeting connexin43 expression accelerates the rate of wound repair. Curr. Biol. *13*, 1697–1703.

51. Bessa, L.J., Fazii, P., Di Giulio, M., and Cellini, L. (2015). Bacterial isolates from infected wounds and their antibiotic susceptibility pattern: some remarks about wound infection. Int. Wound J. *12*, 47–52.

52. Scarpa, E., and Mayor, R. (2016). Collective cell migration in development. J. Cell Biol. *212*, 143–155.

53. Donà, E., Barry, J.D., Valentin, G., Quirin, C., Khmelinskii, A., Kunze, A., Durdu, S., Newton, L.R., Fernandez-Minan, A., Huber, W., et al. (2013). Directional tissue migration through a self-generated chemokine gradient. Nature *503*, 285–289.

54. Venkiteswaran, G., Lewellis, S.W., Wang, J., Reynolds, E., Nicholson, C., and Knaut, H. (2013). Generation and dynamics of an endogenous, self-generated signaling gradient across a migrating tissue. Cell *155*, 674–687.

55. Ito, N., Yokomizo, T., Sasaki, T., Kurosu, H., Penninger, J., Kanaho, Y., Katada, T., Hanaoka, K., and Shimizu, T. (2002). Requirement of phosphatidylinositol 3-kinase activation and calcium influx for leukotriene B4-induced enzyme release. J. Biol. Chem. *277*, 44898–44904.

56. Enyedi, B., Jelcic, M., and Niethammer, P. (2016). The cell nucleus serves as a mechanotransducer of tissue damage-induced inflammation. Cell *165*, 1160–1170.

57. Oates, A.C., Gorfinkiel, N., González-Gaitán, M., and Heisenberg, C.-P. (2009). Quantitative approaches in developmental biology. Nat. Rev. Genet. *10*, 517–530.

58. Sarris, M., Olekhnovitch, R., and Bousso, P. (2016). Manipulating leukocyte interactions in vivo through optogenetic chemokine release. Blood *127*, e35–e41.

59. Tauzin, S., Starnes, T.W., Becker, F.B., Lam, P.Y., and Huttenlocher, A. (2014). Redox and Src family kinase signaling control leukocyte wound attraction and neutrophil reverse migration. J. Cell Biol. *207*, 589–598.

60. Renshaw, S.A., Loynes, C.A., Trushell, D.M.I., Elworthy, S., Ingham, P.W., and Whyte, M.K.B. (2006). A transgenic zebrafish model of neutrophilic inflammation. Blood *108*, 3976–3978.

61. Kwan, K.M., Fujimoto, E., Grabher, C., Mangum, B.D., Hardy, M.E., Campbell, D.S., Parant, J.M., Yost, H.J., Kanki, J.P., and Chien, C.-B. (2007). The Tol2kit: a multisite gateway-based construction kit for Tol2 transposon transgenesis constructs. Dev. Dyn. *236*, 3088–3099.

62. Westerfield, M. (2007). The Zebrafish Book. A Guide for the Laboratory Use of Zebrafish (Brachydanio rerio). Eugene, Oregon, Fifth Edition (University of Oregon).

63. Yoo, S.K., Lam, P.-Y., Eichelberg, M.R., Zasadil, L., Bement, W.M., and Huttenlocher, A. (2012). The role of microtubules in neutrophil polarity and migration in live zebrafish. J. Cell Sci. *125*, 5702–5710.

64. Peters-Golden, M., and Brock, T.G. (2003). 5-lipoxygenase and FLAP. Prostaglandins Leukot. Essent. Fatty Acids *69*, 99–109.

65. Coombs, C., Georgantzoglou, A., Walker, H.A., Patt, J., Merten, N., Poplimont, H., Busch-Nentwich, E.M., Williams, S., Kotsi, C., Kostenis, E., and Sarris, M. (2019). Chemokine receptor trafficking coordinates neutrophil clustering and dispersal at wounds in zebrafish. Nat. Commun. *10*, 5166.

66. Schindelin, J., Arganda-Carreras, I., Frise, E., Kaynig, V., Longair, M., Pietzsch, T., et al. (2012). Fiji: an open-source platform for biological-image analysis. Nat. Methods *9*, 676–682.

STAR★METHODS

KEY RESOURCES TABLE

REAGENT or RESOURCE	SOURCE	IDENTIFIER
Antibodies		
Chicken anti-GFP	Abcam	CAT#ab13970; RRID: AB_300798
Rabbit anti-tRFP	Evrogen	CAT#AB233; RRID: AB_2571743
Rabbit anti-Cx43	Sigma-Aldrich	CAT#C6219-1MG; RRID: AB_476857
Anti-chicken Alexa488	Invitrogen	CAT# A-11039; RRID: AB_142924
Rabbit anti-β-Tubulin	Abcam	CAT#ab209866
Rabbit IgG control	Merck	CAT#12-370; RRID: AB_145841
Goat anti-rabbit HRP antibody	Abcam	CAT#ab97080; RRID: AB_10679808
Anti-rabbit-Cy3	Jackson ImmunoResearch	CAT#711-165-152; RRID: AB_2307443
Bacterial and Virus Strains		
PAO1 *Pseudomonas aeruginosa*	Martin Welch	N/A
Chemicals, Peptides, and Recombinant Proteins		
Cxcl8a	ProteinTech	N/A
non-hydrolysable ATP-γS	Sigma-Aldrich	CAT#A1388-1MG
SKF 96365	Cayman Chemical	CAT#10009312
P. aeruginosa isolation agar	Scientific laboratory supplies LTD	CAT#257002
Nalidixic acid	Scientific laboratory supplies LTD	CAT#SR102E
DMEM	Invitrogen	CAT# 16219961
FBS	GIBCO ThermoFisher Scientific	CAT# 16140071
Penicillin/Streptomycin	Sigma-Aldrich	CAT#TMS-AB2-C
DAPI	Sigma-Aldrich	CAT#D8417-1MG
Lipofectamine 2000	invitrogen	CAT# 11668019
LTB4	Sigma-Aldrich	CAT#L0517-10UG
NF279	BIO-TECHNE LTD	CAT#1199
U-75302	Cayman Chemical	CAT#70705
MS-222	Sigma-Aldrich	CAT# E10521-50G
2-APB	Tocris Bioscience	CAT#1224
Methylene blue	Sigma-Aldrich	CAT# M9140-25G
1-phenyl-2-thiourea	Sigma-Aldrich	CAT#P7629-25G
EGTA	Sigma-Aldrich	CAT#E3889-25G
Carbenoxolone	Sigma-Aldrich	CAT#C4790-5G
Methanol-free formaldehyde	Thermofisher	CAT#28906
Sudan Black	Sigma-Aldrich	CAT#3080-1KT
PBS	Oxoid	CAT#BR0014G
Tween-20	Sigma-Aldrich	CAT#P-1379
Syto-62	Invitrogen	CAT#S11344
Low melting point agarose	Invitrogen	CAT#16520-100
A23187	Sigma-Aldrich	CAT# C7522-10MG
Critical Commercial Assays		
KOD Hot start kit	Novagen, TOYOBO	CAT#71086
SuperScript III Reverse Transcriptase	Invitrogen	CAT#18080051
RNAeasy minikit	QIAgen	CAT#74104
Pierce ECL Plus Western Blotting Substrate	Invitrogen	CAT#32132
Sp6 mMessage mMachine kit	Ambion	CAT#AM1340

(*Continued on next page*)

Continued

REAGENT or RESOURCE	SOURCE	IDENTIFIER
Experimental Models: Cell Lines		
HEK293T cells	Felix Randow's laboratory	N/A
Experimental Models: Organisms/Strains		
Zebrafish Tg(*lyz*:GCamp6F)	This paper	N/A
Zebrafish Tg(*lyz*:*cx43 dn*-T2A-mCherry)	This paper	N/A
Zebrafish Tg(*lyz*:tRFP-5LO)	This paper	N/A
Zebrafish Tg(*mpx*:GFP)^{i114}	[60]	N/A
Zebrafish Tg(*lyz:lta4h*-eGFP)	This paper	N/A
Zebrafish AB strain	PDN fish facility	N/A
Oligonucleotides		
Primer *cx43* Forward: 5¢-GCTCTCCA CTCTTTACTTCTTTCCAG-3¢	This paper	N/A
Primer *cx43* Reverse: 5¢-GTATTGCACTTGAAAGCTGACTGC-3¢	This paper	N/A
Primer *cx43.4* Forward: 5¢-GAGTCGTCATCGCGAGACATTGA-3¢	This paper	N/A
Primer *cx43.4* Reverse: 5¢-GTCTATGAGTCTCAATCAAGCATGGATCC-3¢	This paper	N/A
Primer *lta4h* Forward: 5¢-TCTGAGAAGGAATATGTGGATGAA-3¢	[18]	N/A
Primer *lta4h* Reverse: 5¢-CAGCAAGAGATCTGTCTCCA-3¢	[18]	N/A
cx43 translation morpholino: 5¢-GTTCTAGCTGGAAAGAAGTAAAGAG-3¢	Gene Tools	ZFIN: ZDB-MRPHLNO-080818-1
cx43.4 Splice Morpholino: 5¢-ACTTCTCCATCTCCGTTATATTTTG-3¢	Gene Tools	N/A
Control Morpholino 5¢-CCTCTTACCTCAGTTACAATTTATA-3¢	Gene Tools	https://www.gene-tools.com/content/negative-control-morpholino-oligos
lta4h splice morpholino: 5¢-CAGTCTGATCAAGAGAAAGACTCGA-3¢	Gene Tools	ZFIN: DB-MRPHLNO-171122-3
Recombinant DNA		
GCamp6F	[22]	N/A
lta4h-EGFP	Gene synthesized (Genewiz)	*lta4h cDNA:* ENSDART00000028171.7
tRFP-*5lo*	cDNA library from whole adult zebrafish	5lo/alox5a cDNA: ENSDART00000079884.6
*cx43*dn_T2a_mCherry	(Gene synthesized (Genewiz)	N/A
Cxcl8-mCherry	[30]	N/A
pCS2-TP	[61]	N/A
Software and Algorithms		
MATLAB R2018b	https://www.mathworks.com/	N/A
Imaris v8.2	https://imaris.oxinst.com/	N/A
Fiji	https://fiji.sc/	N/A
Other		
Bolt 10% Bis-Tris Plus Gel	Invitrogen	CAT#NW00100BOX
1mm glass beads	BioSpec	CAT#11079110
iBlot2 transfer stack	Life Technologies	CAT#IB23001

CellPress
OPEN ACCESS

RESOURCE AVAILABILITY

Lead Contact

Further information and requests for resources and reagents should be directed to and will be fulfilled by the Lead Contact, Milka Sarris (ms543@cam.ac.uk).

Materials Availability

Plasmids and zebrafish lines generated in this study are available upon request to the lead contact.

Data and Code Availability

The custom MATLAB codes are available at GitHub [https://github.com/LeukocyteMotionAndDynamics/NeutrophilSwarming] with sample datasets.

EXPERIMENTAL MODEL AND SUBJECT DETAILS

Zebrafish were maintained in accordance with UK Home Office regulations, UK Animals (Scientific Procedures) Act 1986. Adult zebrafish were maintained under project license 70/8255, which was reviewed by the University Biomedical Services Committee. Animals were maintained according to ARRIVE guidelines. Zebrafish were bred and maintained under standard conditions at 28.5 ± 0.5°C on a 14h light: 10h dark cycle. Embryos were collected from natural spawnings at 4-5 hours post-fertilization (hpf) and thereafter kept in a temperature controlled incubator at 28°C. Embryos were grown at 28°C in E3 medium, bleached as described in the Zebrafish Book [62] and then kept in E3 medium supplemented with 0.3 μg/ml of methylene blue and 0.003% 1-phenyl-2-thiourea (Sigma-Aldrich) to prevent melanin synthesis. For live imaging of neutrophils expressing fluorescent markers, methylene blue was omitted from E3 medium to minimize tissue autofluorescence. All embryos were used between 2.5-3.5 dpf, thus before the onset of independent feeding. For live imaging or fixation, larvae were anesthetized in E3 containing 160-200mg/L MS-222 (Sigma). Where indicated, larvae were treated with 50 μM Calcium ionophore A23187 (Sigma), 10 μM NF279 (BIO-TECHNE LTD), 50 μM Carbenoxolone (Sigma), 3μM U-75302 (Cayman Chemical), 20μM SKF 96365 (Cayman Chemical), 25μM 2-APB (Tocris Bioscience), 0.5M EGTA (Sigma) or 1:1000 ethanol in E3.

METHOD DETAILS

DNA constructs and transgenic zebrafish lines

Transgenic Tg(*mpx*:GFP)^{i114} zebrafish originated from the lab of S. Renshaw [60]. The new transgenic lines were made using a backbone DNA construct carrying a Lysozyme C promoter (*lyz*), for neutrophil-specific expression, minimal Tol2 elements, for efficient integration, and a SV40 polyadenylation sequence [63]. The references for the sequences cloned in this backbone vector are:

GCamp6F: cDNA originally described by Chen et al., 2013 [22]
lta4h-EGFP: *lta4h cDNA (*Ensembl: ENSDART00000028171.7) synthesized by Genewiz
tRFP-*5lo*: 5lo/alox5a cDNA (Ensembl: ENSDART00000079884.6) amplified from a cDNA library from whole adult zebrafish
*cx43*dn_T2a_mCherry: cDNA for *cx43*dn described by Omayada et al., 2002 [36] was synthesized by Genewiz

The sequence of zebrafish alox5/5-LO was chosen over 4 alox genes on the basis of similarity with human 5-LO [64]. For transgenesis, 0.5nL of solution containing 25ng/μL DNA plasmid and 35 ng/μL were injected into the cytoplasm of one-cell stage embryos. Transposase mRNA was synthesized from pCS2-TP [61] by *in vitro* transcription (SP6 message machine, Ambion). Injected embryos were stored at 28°C until 5dpf and thereafter were raised in the fish nursery according to standard rearing protocols. At 3 months old, F0 fish were outcrossed to a wild-type (TL) line in order to screen for germline transgenesis.

Morpholino injections

Morpholinos were ordered on GENE TOOLS LTD and their names, sequences, types and origins are indicated below. All morpholinos were injected in one cell stage eggs in a morpholino injection solution (120mM KCl, 20mM HEPES, 0.1% phenol red). We used morpholino against the two isoforms found in neutrophils: cx43 (also called *cx43.3*) [35] and against *cx43.4*. 1nL of 0.2mM of each morpholino was injected (0.4mM total). As injection control we used 0.4mM of Negative Vivo-Morpholino control oligo. For *lta4h* knockdown 3nL of 0.5mM MO *LTA4H* were injected [18]. List of morpholinos injected:

cx43 (Translation-blocking): 5′-GTTCTAGCTGGAAAGAAGTAAAGAG-3′
cx43.4 (Splice-blocking): 5′-ACTTCTCCATCTCCGTTATATTTTG-3′
Standard negative control oligo: 5′-CCTCTTACCTCAGTTACAATTTATA-3′
lta4h (Splice-blocking): 5′-CAGTCTGATCAAGAGAAAGACTCGA-3′

Western blotting

For western blotting, 10 larvae (3dpf) of each genotype were collected. Larvae were then lysed in 100μL of OCG buffer (0.3M NaCl, 2.5μM EDTA pH8, 0.9M Tris HCl pH7.5, protease inhibitors, phosphatase inhibitor) with 1mm glass beads (BioSpec) for 3x20sec in the sonicator Bioruptor (diagenode). Qubit protein assay kit (Invitrogen) was used to obtain protein concentration. Proteins (25μg) were resolved on a Bolt 10% Bis-Tris Plus Gel (Invitrogen), blotted onto nitrocellulose membrane using iBlot2 transfer stacks (Life Technologies) according to manufacturer's protocol. Proteins were probed with rabbit anti-human Cx43 (1:2000) (Sigma-Aldrich) and rabbit anti-β-Tubulin antibodies (1:2000) (Abcam) after saturation in PBT (PBS, 0.1% Tween-20) containing 5% of milk. Proteins were then revealed using an enhanced chemiluminescence detection system (Pierce ECL Plus Western Blotting Substrate, Invitrogen) with goat anti-rabbit HRP antibody (1:2000) (Abcam).

Two-photon laser wound and live imaging

For mechanical ventral fin wounds, larvae were mounted immediately after wound onto a glass-bottom plate in 1% low melting agarose (Invitrogen) or a custom-built coverslip chamber (for when using an upright scope). Agarose-embedded embryos were covered with 2 mL E3 medium (supplemented with MS-222) and imaged either on i) an inverted PerkinElmer UltraVIEW ERS, Olympus IX81 spinning disk confocal microscope with a 30x/1.05 NA silicon (Olympus) or 40x/1.25 NA silicon objective (Olympus) and 488nm for GFP excitation and 561 for tRFP or mCherry or ii) on an upright Nikon E1000 microscope coupled to a Yokogawa CSU10 spinning disc confocal scanner unit with a 20x/0.75 NA air objective (Nikon) or 10x/0.5 NA air objective (Nikon) and illuminated using a Spectral Applied Research LMM5 laser module (491 nm for GFP excitation; 561 nm for Ruby or TagRFP or mCherry). Confocal stacks using a 2μm z-spacing were acquired every 20-40 s.

Laser wounding was performed on a two-photon scanning miscroscope (LaVision Biotec TriM Scope II). A tunable ultrafast laser (Insight DeepSee, SpectraPhysics) was tuned to 930 nm and the laser power adjusted to approximately 500mW. A square region of interest (ROI) of ~40μm in width was defined in one focal plane followed by single laser scan across the ROI at a pixel spacing of 240nm and dwell time of 13 μs. Confocal stacks were acquired immediately after, using a 25x/1.05 NA water-dipping lens. GFP was imaged with 930nm and DsRed was imaged with a 1040nm line. For imaging 5-LO translocation, the resolution of imaging with the two-photon microscope was limiting. Larvae were thus transferred (within 10-20 min) for imaging onto an upright Nikon E1000 microscope coupled to a Yokogawa CSU10 spinning disc confocal scanner unit with a 40x/0.80W water objective (Nikon). In some cases, PI (50μg/ml) was added to the medium 30 min prior to imaging. PI penetration was observed only with superficial laser wound.

For the Cxcl8a response assay, HEK293T cells were cultured in DMEM (Invitrogen) containing 10% FBS (GIBCO ThermoFisher Scientific) and 1% Penicillin/Streptomycin (Sigma). HEK293 cells were transfected with Cxcl8a-mCherry using Lipofectamine-2000 (Invitrogen) (construct described in [30]. Transfected cells were incubated at 37°C (with 5% CO_2) overnight, harvested the following morning and resuspended in DPBS (Invitrogen) at a density of $30x10^6$/ml. Cells were transplanted above the yolk into 48hpf Tg(*lyz*:GCamp6F) larvae as previously described [30]. Validation of Cxcl8a-mCherry secretion and function *in vivo* was described previously [30].

Tail fin wound infection experiments

3dpf larvae were anesthetized with 160-200mg/L MS-222 (Sigma) and their tail fin was amputated using a sterile surgical scalpel blade (Swann-Morton, 23). Larvae were subsequently (within 5 min) incubated for 2 hours at 33°C in Ringer (145mM NaCl, 2mM KCl, 1.5mM K_2HPO_4, 1mM $MgSO_4$, 10mM HEPES, 2mM $CaCl_2$ and 10mM glucose, pH7.2) medium with 1.10^7/mL *P. aeruginosa* PAO1 strain (provided by Dr. Martin Welch). Following incubation, larvae were washed 5 times in PBS and separated in individual wells containing 100μL of PBS on a 96 well plate, to avoid transmission of infection across larvae. Survival and bacterial burden were monitored at 2, 6 and 18 hours post-wounding. For determining the bacterial burden, larvae were homogenized with a pestle gun (Anachem LTD) in 100μL PBS in a 4.5mL Eppendorf tube. Serial dilutions of these homogenates were then plated on *P. aeruginosa* isolation agar (Scientific laboratory supplies LTD) supplemented with cetrimide (200 mg/L) and nalidixic acid (15 mg/L) (CN supplement; Scientific laboratory supplies LTD) and incubated for 24h at 37°C. Colonies were counted to determine the number of colony forming units contained in one fish.

Two-photon imaging of wound colonization by bacteria

P. aeruginosa PAO1 strain were incubated for 30 min with 10μM Syto62 (Invitrogen) in PBS at 37°C. This was followed by four washes by centrifugation in PBS. Larvae were anesthetized with 160-200mg/L MS-222 (Sigma) at 3dpf and mounted in 1% agarose in a custom-made imaging chamber, consisting of a round coverslip above and below the sample sealed onto a metallic ring. Agarose was allowed to set and then was removed around the tail with a glass capillary in order to allow bacterial swimming toward the wound. E3 medium with 1.10^7/mL labeled PAO1 and 160-200mg/L MS-222 was added to the chamber, which in turn was sealed and sterilized on the outside using a 1% virkon solution and 70% ethanol. The fish were subsequently imaged and wounded on a two-photon laser scanning microscope as described above.

Chemoattractant injections in the otic cavity

Morpholino injected and non-morpholino injected 3-dpf larvae were injected in the otic vesicle with 1nL of 30nM LTB4 (Sigma),30nM Cxcl8a (ProteinTech, custom-made) or 200μM non-hydrolysable ATP-γS (Sigma) in combination with 10% phenol red to track the injection. The control solution for LTB4 was ethanol at 1:1000 dilution. The control solution of Cxcl8a and non-hydrolysable

ATP-γS was 0.1% BSA in PBS. Larvae were imaged on a spinning-disc confocal microscope 15 min after injection or fixed for Sudan Black staining 45 min after injection.

Whole-mount Sudan Black staining and immunohistochemistry

Tail-fin amputated larvae or larvae microinjected in the otic cavity were fixed after 3 hours and 45 min respectively in 1ml of 4% ethanol-free formaldehyde (Polysciences, Warrington, PA) in PBS (PBS; Sigma-Aldrich) overnight at 4°C with agitation. Fixed larvae were rinsed in PBT (PBS with 0.1% Tween-20; Sigma-Aldrich) twice for 5 minutes and incubated in 1ml Sudan Black (Sigma-Aldrich) for 15 min. Following staining, larvae were washed in 70% ethanol for several hours and transferred to 30% ethanol overnight at 4°C with agitation. Larvae were washed in PBT for 10 minutes, transferred to increasing concentrations of glycerol and stored in 80% glycerol at 4°C. Larvae were imaged on an optical microscope Stemi 2000-CS (ZEISS) mounted with axiocam ERcs 5 s (Zeiss).

For immunohistochemistry, 3dpf larvae were fixed overnight in 4% formaldehyde (ThermoFisher) at 4°C, washed twice in PBT (PBS, 0.1% Tween-20) and then dehydrated overnight in methanol 100% at −20°C. Larvae were progressively rehydrated by decreasing methanol concentration, heated for 15min at 70°C and fixed in ice cold acetone for 20 min at −20°C. After blocking in 10% sheep serum (Sigma-Aldrich), proteins were probed with primary antibody and revealed using secondary antibody. GFP^+ neutrophils, tRFP-5-LO and Cx43 were detected using chicken anti-GFP (abcam) at 1:500, rabbit anti-tRFP (Evrogen) at 1:500 and rabbit anti-Cx43 (Sigma) at 1:300 respectively. Secondary antibodies used were anti-chicken-Alexa488 (Invitrogen) and anti-rabbit-Cy3 (Jackson) at 1:500. Nuclei were stained with DAPI at 0.5μg/mL (Sigma Aldrich). Subsequently larvae were embedded in 80% glycerol and mounted for confocal observation (Olympus Fluoview FV1000).

RT-PCR of Cx43 genes in neutrophils

RNA extraction: Larvae were snap frozen in liquid nitrogen after removal of E3 medium. RNA was extracted with the RNAeasy minikit (QIAgen) according to manufacturer's instruction. RNA was then reverse transcribed with SuperScript III Reverse Transcriptase (Invitrogen). PCR was performed using the KOD Hot Start DNA polymerase kit (Novagen, TOYOBO).

List of primers used for RT PCR:
cx43 Forward 5′-GCTCTCCA CTCTTTACTTCTTTCCAG-3′
cx43 Reverse 5′-GTATTGCACTTGAAAGCTGACTGC-3′
cx43.4 Forward 5′-GAGTCGTCATCGCGAGACATTGA-3′
cx43.4 Reverse 5′-GTCTATGAGTCTCAATCAAGCATGGATCC-3′
lta4h Forward 5′-TCTGAGAAGGAATATGTGGATGAA-3′
lta4h Reverse 5′-CAGCAAGAGATCTGTCTCCA-3′

Extraction of cell trajectories

Analysis of neutrophil trajectories was performed in Imaris v8.2 (Bitplane AG, Zürich, Switzerland) on 2D maximum intensity projections of the 4D time-lapse videos. For laser-wounded larvae, unless otherwise indicated, trajectories were extracted from a cropped area covering the ventral fin and the part of CHT in which there was neutrophil immobilization. For ventral fin-wounded larvae trajectories were extracted from a cropped area covering the entire ventral fin. A track duration threshold of 3 time-frames was defined to exclude short-lived tracks. Manual track corrections were also applied where needed. Instantaneous (x,y,t) coordinates over time were exported into Microsoft Excel 2016 spreadsheets files (Microsoft Corporation, Redmond, WA).

Extraction of cell surface data

Analysis of neutrophil cluster size and calcium signal was performed in Imaris. Neutrophils were segmented as surfaces and manual surface splitting or merging was applied where needed. Instantaneous neutrophil cluster size and calcium signal intensity were exported into Microsoft Excel 2016 spreadsheets files.

Definition of wound perimeter and of clustering versus migrating cells

For laser wounds, the perimeter of the wound was manually defined in MATLAB R2018b (The MathWorks, Natick, MA) as a set of points surrounding the autofluorescent area of the wound. For mechanical ventral fin wounds, the perimeter of the wound was manually defined in MATLAB as a set of points surrounding the area maximally occupied by neutrophils at the wound (occupied wound area), as indicated by a continuous surface of high intensity on a time-projection of the movie, as previously described [65].

Quantification of GCamp6F levels

Except for Figure 2 (see previous section on 5-LO-translocation analysis), calcium signal values were extracted from Imaris and imported into MATLAB for plotting. For the laser wound experiments, calcium values for individual segmented neutrophils were normalized to the mean calcium value of the neutrophils in the whole area outside the wound, prior to wounding. In all corresponding bar graphs, the first 3-9 frames post-wounding were excluded to eliminate distortion of the neutrophil data by the tissue-scale calcium wave. For mechanical fin wound videos, the calcium values for individual neutrophils were normalized to the mean calcium value of the neutrophils in the whole area outside the wound, at the first time point of imaging. For the analysis of GCamp6F levels in the ear, the mean intensity of cells in the ear was divided by the mean intensity in cells outside the ear.

Quantification of GCamp6F levels with neutrophil cluster size

Neutrophil cluster size and calcium signal values were computed in MATLAB and plotted against each other. A threshold of 60 pixels on the size of detected objects was applied to eliminate false detections.

Calculation of neutrophil radial speed

Radial speed was calculated in MATLAB using the following Equation [8]:

$$u_r = u \times \cos\theta$$

where u is the instantaneous speed of neutrophil between two successive positions and θ is the angle between the vector of the movement and the vector that connects the position with the wound. The angle θ was calculated using the vector between the neutrophil position (centroid) and its nearest point to the wound. When the cosine of θ has value 1, the neutrophil migrates directly toward the wound while when it has value -1, the neutrophil migrates directly away from the wound. To uncover trends in directionality of motion in embryos independently from intra-embryonic variation in speed levels, we used a normalization. Instantaneous speed values for individual neutrophils were divided by the mean instantaneous speed value of the corresponding embryo. Normalized radial speeds were computed with the equation:

$$ur_{norm} = u_{norm} \times \cos\theta$$

Normalized radial speed values were binned every 5 or 7.5 min. For the laser wound experiments, the first 3-9 frames post-wounding were excluded for consistency with the calcium signal calculations.

Detection and scoring of 5-LO translocation in zebrafish neutrophils

Automated detection [56] was not applicable to neutrophils due to the irregular shape of the nucleus and their dynamic movement. We thus used visual inspection of the time-lapse videos on Fiji [66] and representative sample videos were confirmed by two viewers. Only unambiguous translocation events were scored.

Analysis of 5-LO translocation in relation to distance or GCamp6F intensity

Frames in which 5-LO translocation events were detected and thereafter analyzed with MATLAB in an automated fashion. Individual cells were segmented using marker-based watershed segmentation and intensity thresholding. Mean fluorescence intensities of the GCamp6F signal in segmented neutrophils were subsequently computed. For each neutrophil, the fluorescence intensity was normalized to the most fluorescent cell in the corresponding frame to allow pooling of values across embryos with different imaging settings. The wound center was manually inputted and the distance of individual neutrophil centroids from the wound center was automatically computed using a custom MATLAB script.

Analysis of GCamp6F in neutrophil cell-cell contacts and neutrophil contacts with necrotic tissue

Contacts between bright and dim GCamp6F$^+$ neutrophils or contacts with PI$^+$ necrotic cells were counted and classified according to whether a sharp increase of fluorescence was observed in the dim cell upon contact or not. These events were quantified using visual inspection of the time-lapse videos on Fiji. Only unambiguous events were scored.

Evolution of neutrophil GCamp6F levels and speed over time and upon contact with necrotic cells

Individual neutrophils were visually inspected to determine the time-point that they touched the PI-stained necrotic cells. This time-point was considered as the time-point 0. The neutrophils were tracked for 180 s before and after this time-point. Individual neutrophil calcium values were normalized with the calcium value of the first time-point of the track.

Analysis of fluorescence intensity of bacteria over time

Segmentation of bacteria was done in Imaris using surface segmentation in the 3D dataset. The segmentation was manually limited to a volume spanning the z dimension of the wound core, taking also into consideration the wound autofluorescence and the location of the neutrophil cluster. Corrections in tissue drift were performed by changing the tracking area in all 3 dimensions (x,y,z) in time, to allow accurate segmentation of this area. Intensity values were extracted in Imaris. A custom-written MATLAB script was used to normalize values to the fluorescent intensity of all bacteria in the first frame before wounding, and to the maximum initial fluorescent intensity of bacteria in the wound area (usually between 5-10 min post wounding), and plot the mean of different time bins across independent larvae. A contribution of fluorescence photobleaching was excluded by performing similar computations in a region of interest with bacteria outside the larva.

QUANTIFICATION AND STATISTICAL ANALYSIS

All error bars indicate SEM. All p values were calculated with two-tailed statistical tests and 95% confidence intervals. t test (pairwise comparisons) and one-way ANOVA (multiple group comparisons) were performed after distribution was tested for normality otherwise non-parametric tests were performed (Mann-Whitney for two-way comparisons and Kruskal-Wallis with Dunn's post-test for multiple comparisons). Unless otherwise indicated, tests were unpaired. Statistical tests were performed

CellPress
OPEN ACCESS

in Prism8 (GraphPad Software, La Jolla, CA). The statistical test and the n number are indicated in the figure legends. The error bars show standard error of the mean across individual embryos or cells except for Figures 2E and 2F where error bars represent 95% confidence intervals of the median. Where the distribution was verified as normal, outliers were removed by applying Rout test. Live imaging experiments were acquired in minimum three independent experiments. In figure panels, * corresponds to $p < 0.03$, ** to $p < 0.002$ and *** to $p < 0.0002$. In line graphs with binned data, * is indicated for the bin with smallest detectable significant difference.

Current Biology, Volume *30*

Supplemental Information

Neutrophil Swarming in Damaged Tissue Is Orchestrated by Connexins and Cooperative Calcium Alarm Signals

Hugo Poplimont, Antonios Georgantzoglou, Morgane Boulch, Hazel A. Walker, Caroline Coombs, Foteini Papaleonidopoulou, and Milka Sarris

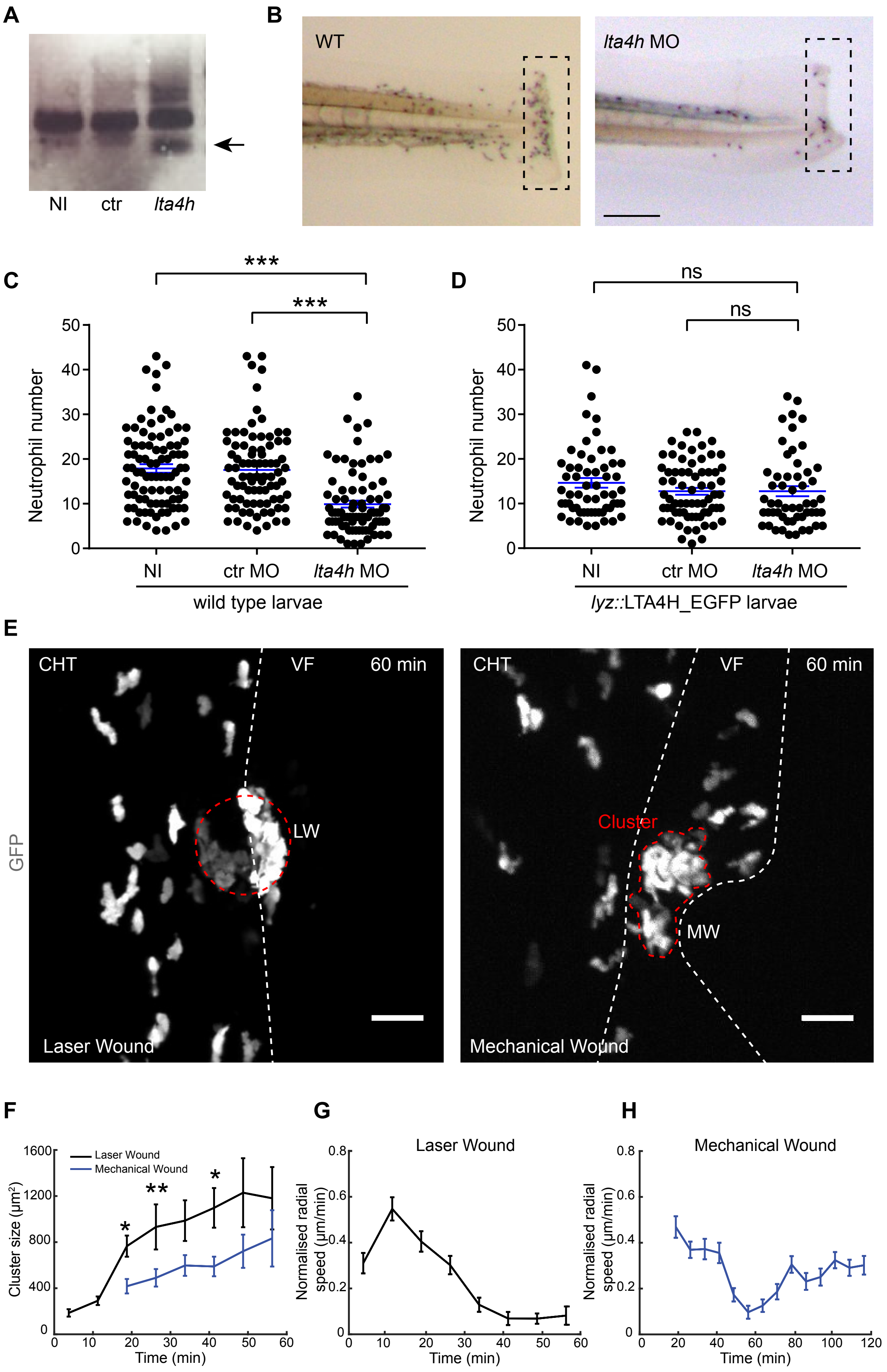
A
NI
ctr
lta4h
B
WT
lta4h MO
C
***
***
Neutrophil number
NI
ctr MO
lta4h MO
wild type larvae
D
ns
ns
Neutrophil number
NI
ctr MO
lta4h MO
lyz::LTA4H_EGFP larvae
E
CHT
VF
60 min
LW
Laser Wound
Cluster
MW
Mechanical Wound
GFP
F
Laser Wound
Mechanical Wound
Cluster size (µm²)
Time (min)
G
Laser Wound
Normalised radial speed (µm/min)
Time (min)
H
Mechanical Wound
Normalised radial speed (µm/min)
Time (min)

Figure S1. Role of neutrophil biosynthesis of LTB4 and dynamics of neutrophil swarming in zebrafish wounds, related to Figure 1.

A RT-PCR of *lta4h* from mRNA extracted from 3 dpf wild type (AB) zebrafish. The arrow denotes the presence of an alternative transcript in larvae injected with a splice-blocking *lta4h* morpholino; NI = Non-injected, Ctr = Control morpholino, *lta4h* = injected with the splice-blocking *lta4h* morpholino.

B Sudan black staining of neutrophils in wild type (WT) and *lta4h* morpholino-injected 3dpf larvae amputated with a scalpel and fixed 3h post-wounding. The dotted lines represent the area in which neutrophils were counted. Scale bar = 100μm.

C, **D** Quantification of the total number of neutrophils recruited to wounds. NI = Non-injected, Ctr MO = Control morpholino, *lta4h* = injected with the splice-blocking *lta4h* morpholino. **C** 81-89 larvae per group pooled from 3 experiments. **D** 54-68 larvae per group pooled from 3 experiments. p value <0.0001. Kruskal-Wallis test with Dunn's post-test.

E Projections from two photon (left) and spinning disk confocal images (right) showing neutrophils from Tg(*mpx*:GFP)^{i114} larvae migrating from the caudal hematopoietic tissue (CHT) towards a laser wound (LW; dotted line) at the ventral fin-CHT boundary (VF/CHT) or mechanical ventral fin wound (MW) respectively. The red dotted line indicates area occupied by the neutrophil cluster. Scale bar = 25μm.

F Evolution of cluster size over time post wounding for neutrophils in Tg(*mpx*:GFP)^{i114} larvae. n=4 larvae with laser wound, n=14 larvae with mechanical fin wound from 2 and 7 experiments respectively. Mann Whitney test.

G Evolution of neutrophil radial speed over time post laser wounding (LW) for neutrophils in Tg(*mpx*:GFP)^{i114} or Tg(*lyz*:GCamp6F) larvae. Instantaneous speed values for individual neutrophils were divided by the mean instantaneous speed value of the corresponding embryo to normalise differences in speed across embryos (see also methods). Error bars represent

standard error of the mean from data binned every 7.5 min. n=1201-1719 cell-steps per bin from n=10 larvae.

H Evolution of neutrophil radial speed over time post mechanical fin wounding for neutrophils in Tg(*mpx*:GFP)^{i114} or Tg(*lyz*:GCamp6F) larvae. Error bars represent standard error of the mean from data binned every 7.5 min from n=1248-1800 cell-steps per bin from 14 larvae.

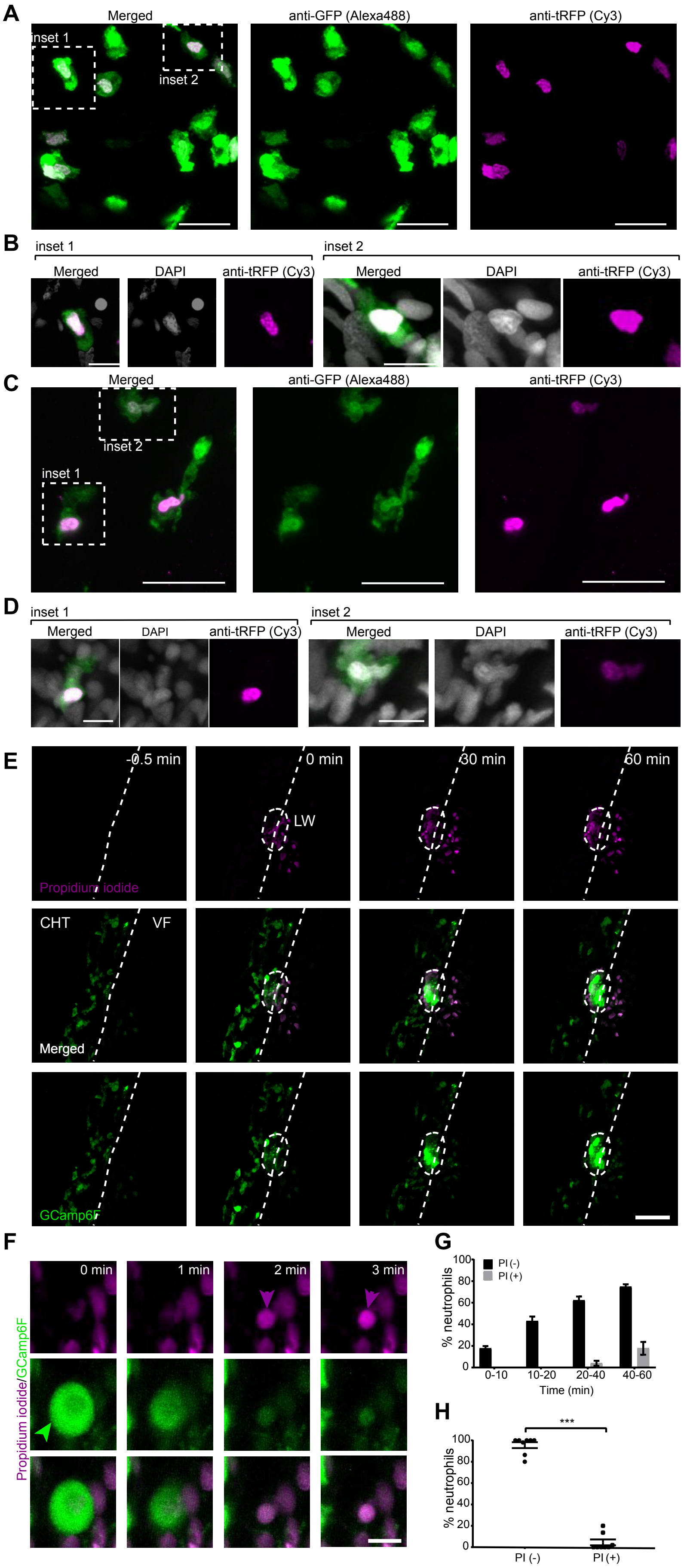

A
Merged
anti-GFP (Alexa488)
anti-tRFP (Cy3)
inset 1
inset 2
B
Merged
DAPI
C
D
E
-0.5 min
0 min
30 min
60 min
LW
Propidium iodide
CHT
VF
GCamp6F
F
1 min
2 min
3 min
Propidium iodide/GCamp6F
G
PI (-)
PI (+)
% neutrophils
0-10
10-20
20-40
40-60
Time (min)
H
***

Figure S2. Nuclear localisation of 5-LO in zebrafish neutrophils and kinetics of neutrophil death in the first hour post-wounding, related to Figures 2 and 3

A-D Laser scanning confocal projections of Tg(*mpx*:GFP)^{i114}xTg(*lyz*:tRFP-5LO) larvae stained with antibodies against GFP (green) and tRFP (magenta) and co-labelled with nuclear DAPI staining (white) A and C represent examples from two different larvae (Scale bar=25µm). B and D represent zoomed-in images corresponding to insets in A and C respectively (Scale bar=10µm).

E Time-lapse series of two-photon confocal projections images of neutrophils Tg(*lyz*:GCamp6F) larvae (green) incubated in PI (magenta). Time post-wounding is indicated in minutes. Dotted line indicates laser wound (LW). CHT: caudal hematopoietic tissue. VF: ventral fin. Scale bar=50µm.

F Zoomed in example of an apoptotic neutrophil. The green arrow indicates a neutrophil with an apoptotic shape in the GCamp6F channel. The magenta arrows indicate the nucleus of this neutrophil subsequently up taking PI. Scale bar = 10µm.

G,**H** Percentage of dead or alive neutrophils over time (**G**) or averaged through the first hour post-wounding (**H**). Data are means of 8 larvae in 7 independent experiments. Mann-Whitney test.

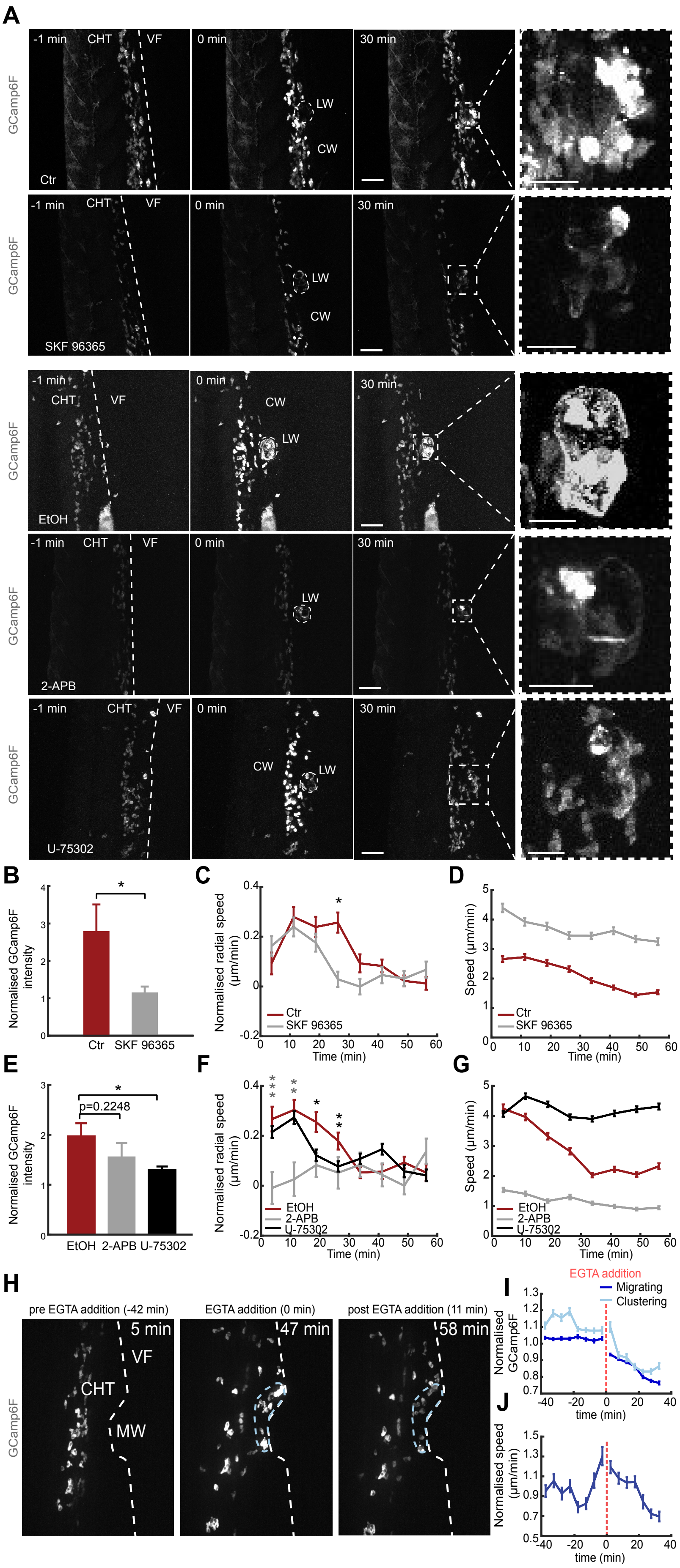
A
-1 min
CHT
VF
0 min
30 min
LW
CW
GCamp6F
Ctr
SKF 96365
EtOH
2-APB
U-75302
B
Normalised GCamp6F intensity
Ctr
SKF 96365
C
Normalised radial speed (µm/min)
Time (min)
D
Speed (µm/min)
E
p=0.2248
EtOH
2-APB
U-75302
F
G
H
pre EGTA addition (-42 min)
EGTA addition (0 min)
post EGTA addition (11 min)
5 min
47 min
58 min
MW
I
EGTA addition
Migrating
Clustering
Normalised GCamp6F
time (min)
J
Normalised speed (µm/min)

Figure S3. Effect of SKF96365, 2-APB, U-75302 and EGTA on neutrophil dynamics, related to Figure 4

A Time-lapse sequence of two-photon confocal image projections showing neutrophils in Tg(*lyz*:GCamp6F)xTg(*lyz*:tRFP-5LO) larvae, without treatment (ctr) or in the presence of 20 µM SKF96365, 25 µM 2-APB, 3 µM U-75302 (and control dilutions of Ethanol to be compared with the latter two). Scale bars = 50µm and 20µm, for zoomed-out and zoomed-in images respectively. CW: calcium wave. Time after laser wounding (LW) is shown in minutes. CHT: caudal hematopoietic tissue. VF: ventral fin.

B Normalised GCamp6F levels in control or SKF96365-treated larvae. n=5 control, n=4 SKF96365-treated larvae from 3 experiments. GCamp6F intensity was normalised as in Figure 1D. Mann-Whitney test.

C-D Neutrophil radial speed (C) or speed (D) over time post-laser wounding in control or SKF96365-treated larvae. n=1226-1527 cell-steps per bin from 5 control larvae and n=968-1219 cell-steps per bin from 4 SKF96365-treated larvae from 3 experiments. Mann-Whitney test.

E Normalised GCamp6F levels in Ethanol control, 2-APB and U-75302-treated larvae. n=5 control, n=4 2-APB-treated larvae and n=8 U-75302-treated larvae from 2, 2 and 3 independent experiments respectively. One-way ANOVA with Dunnet's multiple comparison post-test.

F-G Neutrophil radial speed (F) or speed (G) over time post-laser wounding in Ethanol control, 2-APB and U-75302-treated larvae. n=1464-1743 cell-steps per bin from 5 control larvae, n=679-771 cell-steps per bin from 4 2-APB-treated larvae and n=2712-3135 cell-steps per bin from 8 U-75302-treated larvae from 2, 2 and 3 independent experiments respectively. Kruskal-Wallis with Dunn's multiple comparison test (black stars; ctr vs U-75302, grey stars; ctr vs 2-APB).

H Spinning-disk confocal projection images from neutrophils in Tg(*lyz*:GCamp6F)xTg(*lyz*:tRFP-5LO) post-mechanical fin wound (MW) and EGTA treatment. Time after wounding is indicated within images. Time after EGTA addition is indicated above. The light blue dotted line indicates area occupied by the neutrophil cluster. Scale bar = 50µm.

I Normalised GCamp6F levels in clustering cells (light blue line; corresponding to cells within the wound area occupied by clustering neutrophils shown in J) or migrating cells (dark blue line; corresponding to cells outside the wound area) over time. Red dotted line indicates time of EGTA addition. n=288-810 cell-steps per bin for migrating cells, n=40-144 cell-steps per bin for clustering cells from 5 larvae in 2 independent experiments.

J Normalised neutrophil speed in migrating cells over time. Red dotted line indicates time of EGTA addition. n=170-677 cell-steps per bin from 5 larvae in 2 independent experiments.

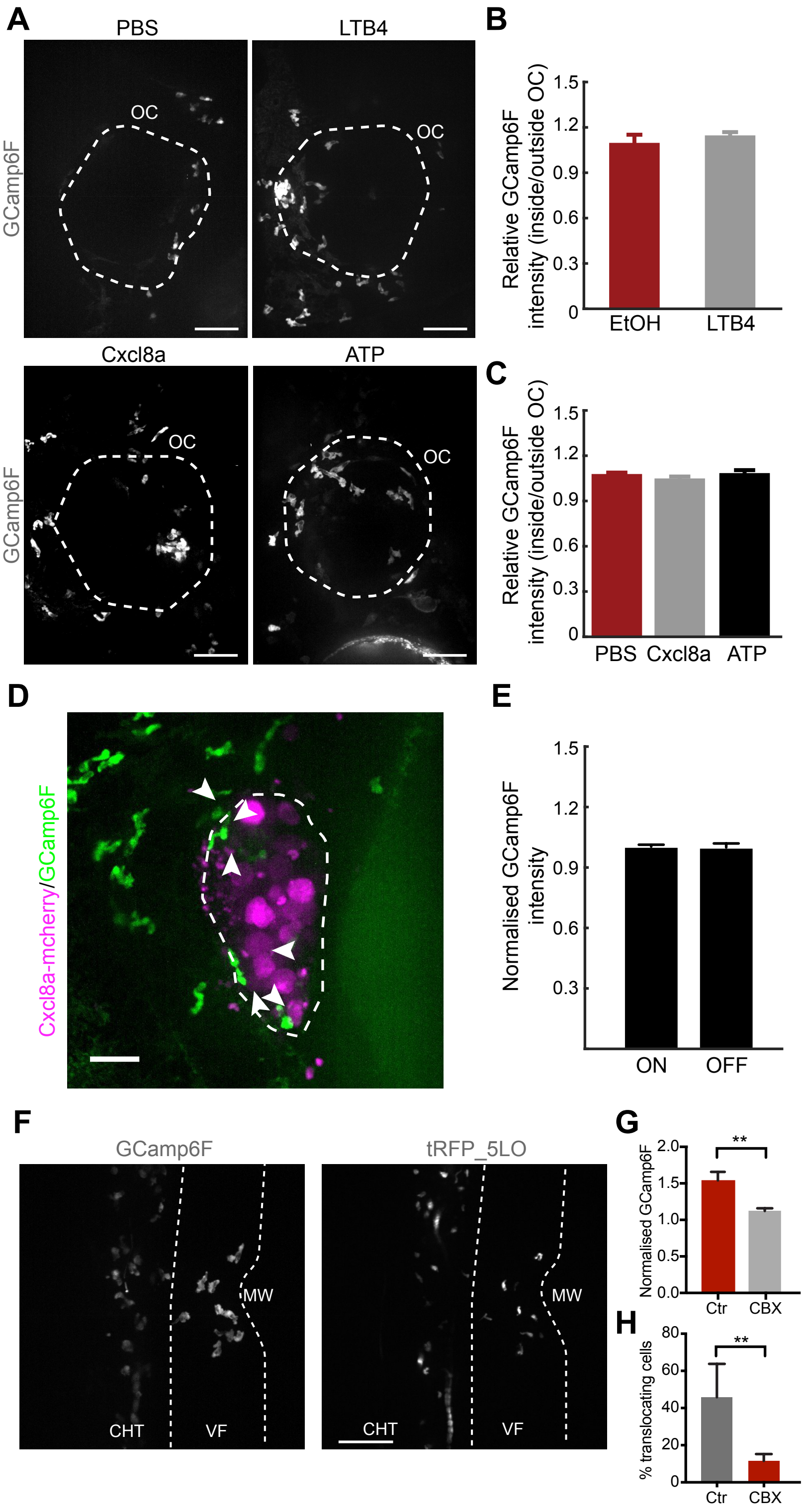
A
PBS
LTB4
Cxcl8a
ATP
OC
GCamp6F
B
Relative GCamp6F intensity (inside/outside OC)
1.5
1.2
0.9
0.6
0.3
0
EtOH
LTB4
C
PBS
Cxcl8a
ATP
D
Cxcl8a-mchery/GCamp6F
E
Normalised GCamp6F intensity
ON
OFF
F
GCamp6F
tRFP_5LO
CHT
VF
MW
G
Normalised GCamp6F
2.0
1.5
1.0
0.5
0.0
Ctr
CBX
**
H
% translocating cells
80
60
40
20
0
Ctr
CBX
**

Figure S4. Effect of chemoattractant injection or transplantation of chemokine-secreting cells on neutrophil calcium dynamics, related to Figures 4 and 5

A Confocal spinning disk projection images of neutrophils in Tg(*lyz*:Gcamp6F) around the otic cavity (OC) of 3 dpf larvae 50 min after local injection of 30 nM LTB4, 30 nM Cxcl8a, 200 µM non-hydrolysable ATP-γS and control and PBS control. The otic cavity is delimited by dotted lines. Scale bar = 50µm.

B and **C** Relative GCamp6F levels in Ethanol (EtOH) control, PBS control, LTB4-injected Cxcl8a-injected and ATP-injected Tg(*lyz*:GCamp6F) larvae. Levels of GCamp6F in neutrophils within the otic cavity (OC) are expressed relative to the levels of cells outside the wound cavity in the first image of the movie. n=5 PBS, n=5 EtOH, n=4 LTB4, n=5 ATP and n=5 Cxcl8a larvae from 2, 3, 2, 2 and 2 independent experiments respectively.

D Confocal spinning disk projection images of neutrophils in Tg(*lyz*:Gcamp6F) 3 dpf larvae (in green) moving around a transplant of HEK293T cells expressing Cxcl8a-mCherry (magenta). The transplantation zone is delimited by dotted lines and neutrophils within this area are indicated with white arrows. Scale bar = 25µm.

E Quantification of GCamp6F intensity in neutrophils on the Cxcl8a transplant (ON) and beyond (OFF). Data are means of n=4 independent embryos.

F Spinning-disk confocal projection images of neutrophils in Tg(*lyz*:GCamp6F)xTg(*lyz*:tRFP-5LO) larvae 90 minutes after mechanical ventral fin wound (MW) in the presence of 50 µM CBX. Representative control shown in Figure 4F, for comparison. MW: mechanical wound. CHT: caudal hematopoietic tissue. VF: ventral fin. Scale bar = 50µm.

G Mean normalised GCamp6F intensity in Tg(*lyz*:GCamp6F)xTg(*lyz*:tRFP-5LO) larvae treated or not with CBX. GCamp6F intensity was normalised as in Figure 1D and E. Data are

means from n=9 control larvae and n= 5 CBX-treated larvae from 3 and 2 imaging sessions respectively. Mann-Whitney test.

H Percent of translocating neutrophils out of all neutrophils recruited into the ventral fin over 90 min of imaging starting 10 min post-wounding. Data are means of n=7 control larvae and n=5 CBX-treated larvae from 3 and 2 imaging sessions respectively. Mann-Whitney test.

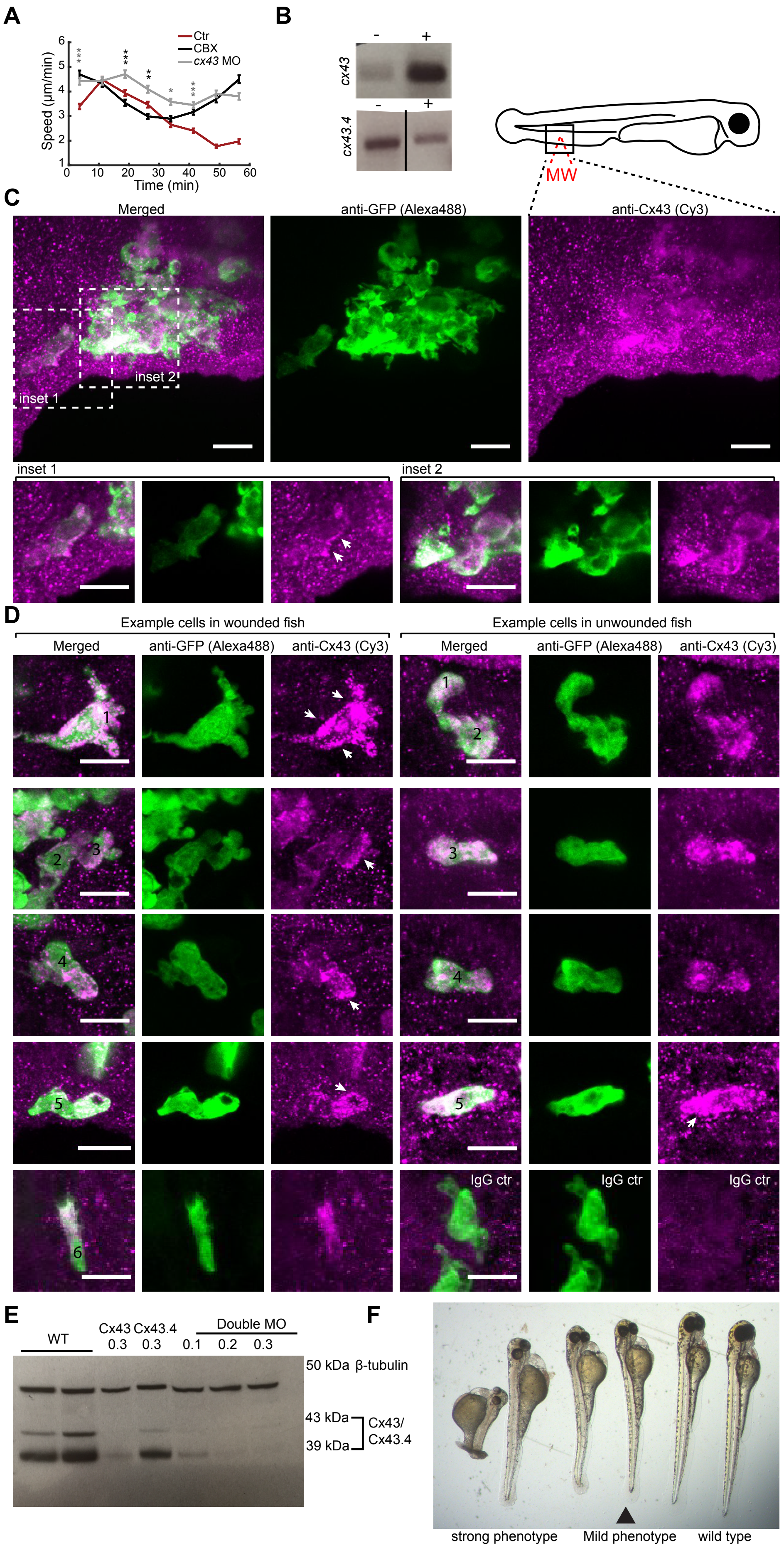
A
Ctr
CBX
cx43 MO
Speed (μm/min)
Time (min)
B
cx43
cx43.4
MW
C
Merged
anti-GFP (Alexa488)
anti-Cx43 (Cy3)
inset 1
inset 2
D
Example cells in wounded fish
Example cells in unwounded fish
Merged
anti-GFP (Alexa488)
anti-Cx43 (Cy3)
IgG ctr
E
WT
Cx43 Cx43.4
0.3 0.3
Double MO
0.1 0.2 0.3
50 kDa β-tubulin
43 kDa
39 kDa
Cx43/
Cx43.4
F
strong phenotype
Mild phenotype
wild type

Figure S5. Neutrophil Cx43 expression and knockdown in zebrafish larvae and effects on neutrophil dynamics, related to Figure 5

A Neutrophil speed over time post-laser wounding in control, CBX-treated and *cx43* MO-treated larvae from Tg(*mpx*:GFP)^{i114} and Tg(*lyz*:GCamp6F) larvae. n=1201-1719 cell-steps per bin from 12 control, n=1268-1535 cell-steps per bin from 8 CBX-treated and n=1308-1554 cell-steps per bin from 11 *cx43* MO-injected larvae from 8, 3 and 4 experiments respectively. Mann-Whitney test (grey stars indicate significance for both Ctr vs CBX and Ctr vs *cx43* MO; black stars indicate significance for just Ctr vs CBX).

B RT-PCR for *cx43* and *cx43.4* expression from cDNA samples of FACS-sorted GFP^{+} (+) or GFP^{-} cells (-) from Tg(*mpx*:GFP)^{i114} 4.5 dpf larvae.

C-D Laser scanning confocal projections of Tg(*mpx*:GFP)^{i114} larvae stained with antibodies against GFP (green) and Cx43/Cx43.4 (magenta). **C** represents example of a cluster overview from one larva (Scale bar = 25µm) with insets showing a closer view of a subset of clustering cells (Scale bar = 10µm). **D** shows additional examples of neutrophils from 5 wounded and 4 unwounded larvae stained with anti-Cx43 and from 1 wounded larva stained with IgG ctr. Scale bar=10µm. Neutrophils are marked with a number. In the examples from wounded larvae, cells 1-5 are at the wound margin and cell 6 is in the CHT. In the examples of unwounded larvae, cells 1-5 are in the CHT. The IgG control example is at the wound margin of a wounded larva. Cartoon shows area of mechanical wounding (MW) and imaging. Arrows indicate example regions with Cx43 stain on the periphery of the neutrophil.

E Western Blot showing the expression of Cx43 and Cx43.4 in 3dpf larvae of wild type AB (WT) and morphant zebrafish larvae. Amount injected for each morpholino in pmol is shown.

F Image of different phenotypes of 3dpf zebrafish larvae injected with *cx43*/*cx43.4* combination MOs. The black arrow indicates the mildest phenotype with detectable difference in eye size that was selected for subsequent experiments.

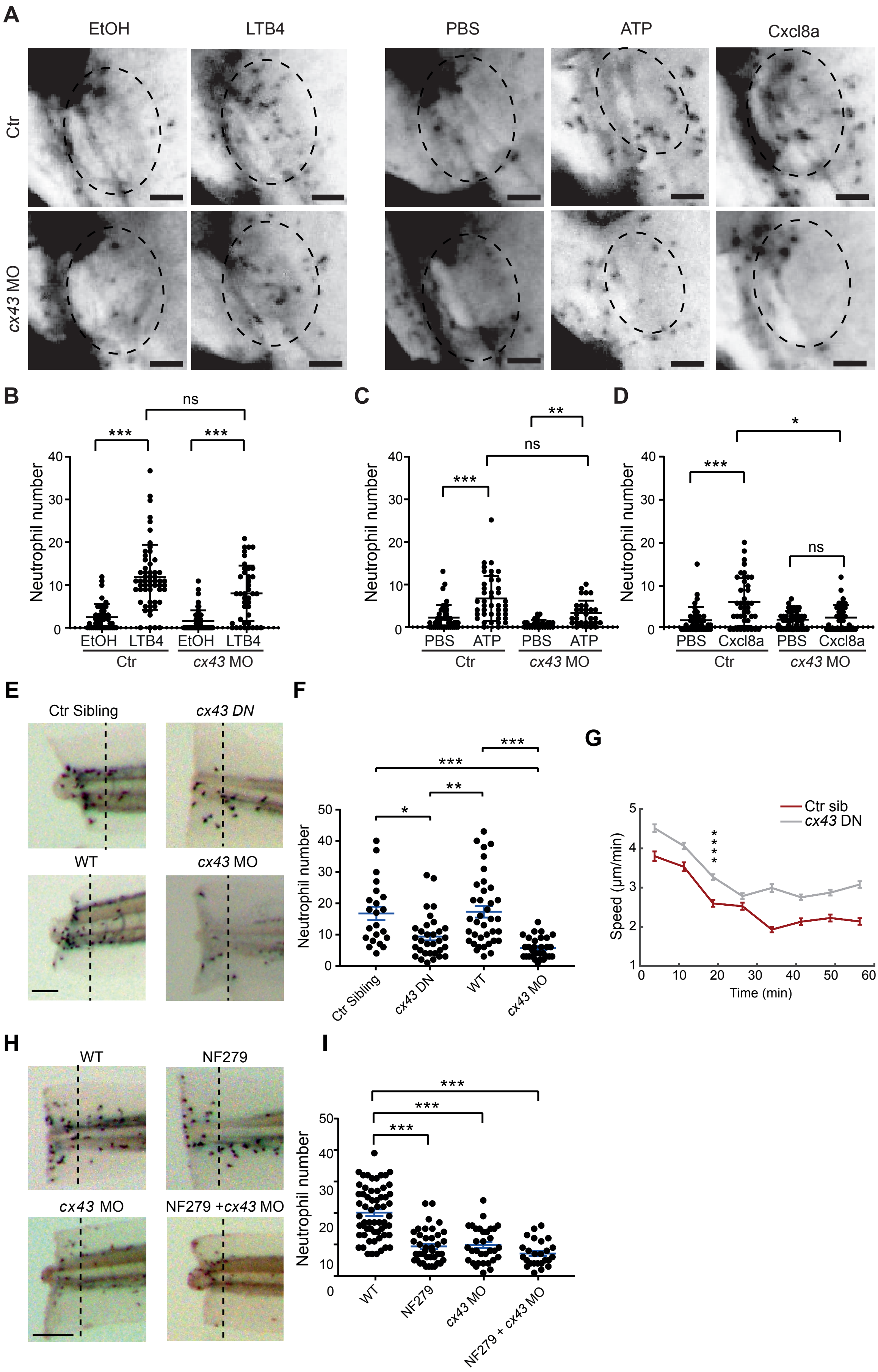
A
EtOH
LTB4
PBS
ATP
Cxcl8a
Ctr
cx43 MO
B
C
D
Neutrophil number
ns
***
**
*
EtOH LTB4 EtOH LTB4
PBS ATP PBS ATP
PBS Cxcl8a PBS Cxcl8a
Ctr
cx43 MO
E
Ctr Sibling
cx43 DN
WT
cx43 MO
F
Ctr Sibling
cx43 DN
WT
cx43 MO
G
Ctr sib
cx43 DN
Speed (μm/min)
Time (min)
H
WT
NF279
cx43 MO
NF279 +cx43 MO
I
WT
NF279
cx43 MO
NF279 + cx43 MO

Figure S6. Recruitment of neutrophils in response to chemoattractant injection and effect of Cx43 inhibition on wound recruitment with or without co-inhibition of ATP signalling, related to Figure 5

A Sudan black staining of neutrophils in transgenic wild type AB larvae 45 min following injection of Ethanol control, PBS control, 30 nM LTB4, 30 nM Cxcl8a and 200 µM non-hydrolysable ATP-γS in the otic vesicle, with or without *cx43* MO injection. The dotted lines represent the periphery of the cavity where neutrophils were counted. Scale bar = 50µm.

B-D Number of neutrophils recruited to the otic vesicle at 45 min. n=30-56 larvae per condition, across 2 independent experiments. Kruskal-Wallis test with Dunn's post-test.

E Sudan black staining of neutrophils in transgenic Tg(*lyz:cx43DN*-T2A-mCherry) larvae (*cx43DN*) and their negatively screened siblings (Sibling) or larvae injected with *cx43/cx43.4* combination of morpholinos (*cx43* MO), fixed at 3h after wounding. The dotted lines represent the area in which neutrophils were counted. Scale bar = 50µm.

F Number of neutrophils recruited to wounds in the different conditions pooled from three independent experiments. *cx43 DN,* n=32 larvae; negative siblings (Ctr sibling), n=22 larvae; WT, n=37 larvae; *cx43* MO, n=30 larvae. Kruskal-Wallis test with Dunn's post-test.

G Neutrophil speed over time post-laser wounding in Tg(*lyz*:GCamp6F)xTg(*lyz*:*cx43DN*-T2A-mCherry) zebrafish larvae, positive (*cx43* DN) or negative for the Cx43 DN-T2A-mCherry transgene (control siblings: Ctr sib). n=1330-1602 cell-steps per bin from 5 control siblings, n=2573-3050 cell-steps per bin from 7 *Cx43* DN larvae from 3 experiments. Mann-Whitney test.

H Sudan black staining of neutrophils in wild type larvae injected or not with *cx43/cx43.4* combination of morpholinos (*cx43* MO) and treated or not with 10µM NF279 and fixed 3h after wounding. Scale bar =100µm.

I Quantification of neutrophil number at wounds in the different conditions. Data pooled from 2 independent experiments. WT (non-injected and not treated with drug), n=60 larvae; NF279, n=40; cx43 MO, n=32; cx43 MO with NF279, n=25. One-way ANOVA with Tukey's multiple comparison test.

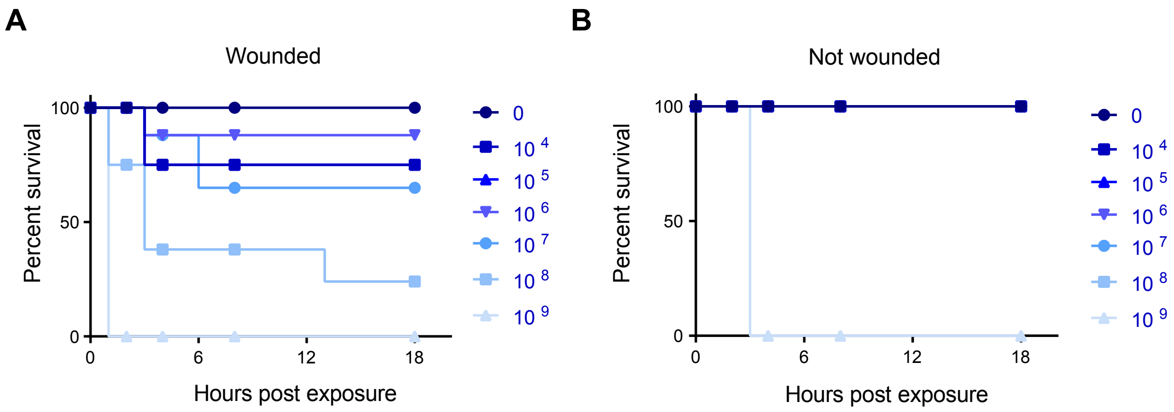

Figure S7. Zebrafish tail fin wound colonisation assay with *P. aeruginosa*, related to Figure 7

A 3 dpf larvae were injured at the tail fin with a scalpel in the presence of titrating doses of *P. aeruginosa* in the bath. Concentration-dependent effects on survival in wounded larvae are shown.

B Same titration in non-wounded larvae. Injury-independent death is observed only at doses below 10^9 CFU/ml. Data represent one experiment with 20 larvae per condition.
